# The Oxidative Stress and Nervous Distress Connection in Gastrointestinal Disorders

**DOI:** 10.3390/biom13111586

**Published:** 2023-10-27

**Authors:** Rhian Stavely, Leah C. Ott, Niloufar Rashidi, Samy Sakkal, Kulmira Nurgali

**Affiliations:** 1 Department of Pediatric Surgery, Massachusetts General Hospital, Harvard Medical School, Boston, MA 02114, USA; 2 Institute for Health and Sport, Victoria University, St Albans, VIC 3021, Australia; 3 Department of Medicine Western Health, The University of Melbourne, St Albans, VIC 3021, Australia; 4 Regenerative Medicine and Stem Cell Program, Australian Institute for Musculoskeletal Science (AIMSS), St Albans, VIC 3021, Australia

**Keywords:** oxidative stress, reactive oxygen species, gastrointestinal, enteric nervous system, enteric neuron, neurointestinal

## Abstract

Oxidative stress is increasingly recognized as a central player in a range of gastrointestinal (GI) disorders, as well as complications stemming from therapeutic interventions. This article presents an overview of the mechanisms of oxidative stress in GI conditions and highlights a link between oxidative insult and disruption to the enteric nervous system (ENS), which controls GI functions. The dysfunction of the ENS is characteristic of a spectrum of disorders, including neurointestinal diseases and conditions such as inflammatory bowel disease (IBD), diabetic gastroparesis, and chemotherapy-induced GI side effects. Neurons in the ENS, while essential for normal gut function, appear particularly vulnerable to oxidative damage. Mechanistically, oxidative stress in enteric neurons can result from intrinsic nitrosative injury, mitochondrial dysfunction, or inflammation-related pathways. Although antioxidant-based therapies have shown limited efficacy, recognizing the multifaceted role of oxidative stress in GI diseases offers a promising avenue for future interventions. This comprehensive review summarizes the literature to date implicating oxidative stress as a critical player in the pathophysiology of GI disorders, with a focus on its role in ENS injury and dysfunction, and highlights opportunities for the development of targeted therapeutics for these diseases.

## 1. Introduction

Oxidative stress is involved in the pathophysiology of a diverse spectrum of gastrointestinal (GI) disorders. These conditions include chronic infections, inflammatory disease, malignancies, diabetes mellitus, ischemia–reperfusion injury, GI toxicities arising from chronic alcohol consumption, radiotherapy, and nonsteroidal anti-inflammatory and chemotherapeutic agents. Many of these GI conditions also exhibit concurrent disruption to the enteric nervous system (ENS). This can manifest as enteric neuropathy, alterations in innervation, neurochemical expression, signaling properties, and neuroinflammation culminating in disrupted GI functions orchestrated by the ENS. These complications can range from impaired movement of luminal contents to more severe and potentially life-threatening issues like impaction and perforation. In this review, we summarize the evidence to date implicating oxidative stress in the pathophysiology of various GI conditions. We highlight the frequency of comorbid enteric neuropathies in GI diseases associated with oxidative stress and discuss preclinical studies that have investigated the mechanisms behind this phenomenon.

## 2. Oxidative Stress 

Cellular organisms harness oxygen’s reactivity to generate substantial energy, vital for sustaining the intricate multicellular lifeforms existing today. This oxidative potential, while advantageous for cellular metabolism and oxidative phosphorylation, also poses risks to cellular structures, necessitating antioxidant defense mechanisms for survival [1,2]. The resultant dualistic nature of oxygen and its reactive oxygen species (ROS) derivatives has rendered reduction-oxidative (redox) equilibrium pivotal in regulating diverse cellular processes [3,4]. Although ROS and reactive nitrogen species (RNS) are commonly acknowledged as agents of cellular impairment, they are concurrently integrated into normative physiological functions. At moderate levels, they mediate apoptosis, intracellular signaling cascades, transcriptional processes, oxygen sensing, and smooth muscle tone [2]. The immune system harnesses ROS for bactericidal properties via enzymatic reactions by leukocytes [5,6,7]. This becomes notably germane within the GI tract, which accommodates approximately 100 trillion commensal bacteria in humans. Nitric oxide (NO), generated enzymatically by three distinct nitric oxide synthase (NOS) isoforms, exemplifies a molecule deeply intertwined with diverse physiological functions. Particularly within the gastrointestinal milieu, enteric neurons wield NO as a neurotransmitter to facilitate smooth muscle relaxation, pivotal for luminal content propulsion along the digestive tract [8].

**Figure 1:**
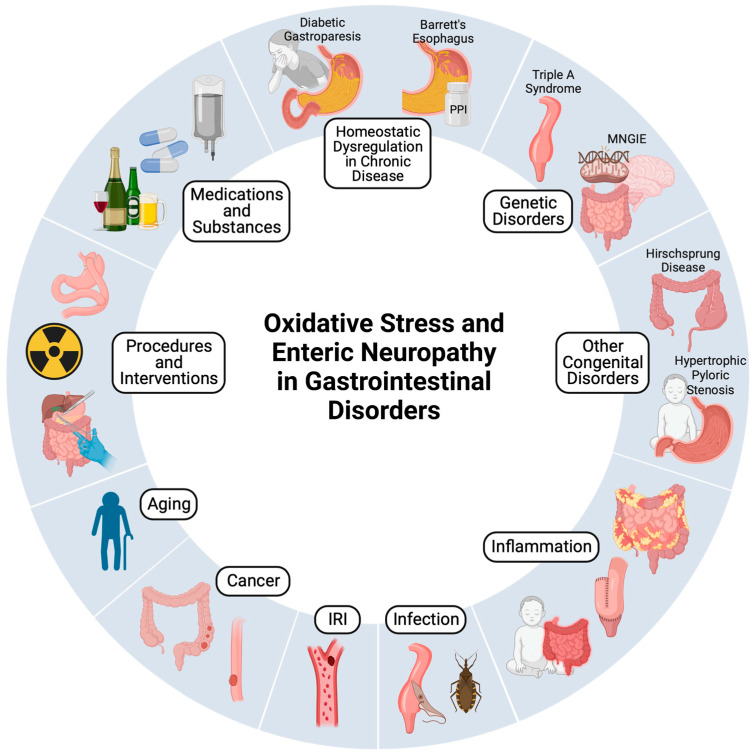
Summary of gastrointestinal (GI) disorders associated with oxidative stress and damage to the ENS. Drug-related GI disorders primarily due to oxidative stress include GI side effects of chemotherapy, gastric ulcers from nonsteroidal anti-inflammatory drug (NSAID) use, and GI sequelae of chronic alcohol use and fetal alcohol syndrome. Oxidative stress may also be induced by procedures and interventions, such as bowel resection with primary anastomosis and radiation therapy. Other acquired disorders that primarily involve oxidative stress include physiological aging (constipation, incontinence), GI cancers (colorectal cancer, esophageal adenocarcinoma), enteritis (necrotizing enterocolitis, pouchitis following ileal pouch–anal anastomosis (IPAA)), and GI ischemia–reperfusion injury (IRI). Congenital GI disorders with oxidative stress as a component of their pathophysiology include genetic disorders (Triple-A syndrome with esophageal achalasia, mitochondrial neurogastrointestinal encephalopathy (MNGIE)) and other congenital disorders (pyloric stenosis, Hirschsprung disease). Acquired GI disorders in which oxidative stress drives disease progression include gastroparesis in diabetes mellitus (primarily caused by hyperglycemia), Barrett’s esophagus (primarily caused by acid exposure), Chagas achalasia (primarily caused by *Trypanosoma cruzi* infection), postoperative ileus (multifactorial, with primary factors including inflammation), and inflammatory bowel disease (IBD, driven by inflammation).

Disruption of this delicate redox balance towards a pro-oxidative milieu culminates in oxidative stress whereby ROS, directly or indirectly, inflict structural alterations to lipids, proteins, and DNA, triggering cellular damage and inflammation [9,10]. The genesis of oxidative stress originates from either a perturbation in ROS/RNS production or their neutralization by antioxidants. Nicotinamide adenine dinucleotide phosphate (NADPH) oxidase (NOX) and xanthine oxidase (XOD) are the primary sources of superoxide (O_2_^.−^) radicals [11]. Other notable ROS and RNS include the hydroxyl radical (•OH), hydrogen peroxide (H_2_O_2_), hypochlorous acid (HOCl), nitric oxide (NO), and peroxynitrite (ONOO-), which fulfill crucial roles in immune responses, albeit excessive production leads to cellular dysfunction and/or cell death [4]. Immune cells generate ROS/RNS via enzymes such as NOX, inducible nitric oxide synthase (iNOS), and myeloperoxidase (MPO), which are pivotal in the normal immune response yet deleterious under prolonged inflammation [5,12,13]. Moreover, excessive O_2_^.−^ produced within the electron transport chain (ETC) complexes of the mitochondria can inflict damage and induce programmed cell death, especially when reacting with NO to form ONOO- [14,15]. Mitochondrial detoxification of O_2_^.−^ to H_2_O_2_ is facilitated by superoxide dismutase (SOD) [13]. However, H_2_O_2_ may also emanate from diverse metabolic processes and dual oxidases (DUOX) [16]. While relatively more stable than O_2_^.−^, the susceptibility of H_2_O_2_ to react with metals, such as Fe^2+^ via the Fenton reaction, renders its detoxification by catalase (CAT) imperative [17]. The glutathione system, encompassing glutathione peroxidase and glutathione reductase, further amplifies the cellular arsenal against excessive ROS accumulation [18]. The gut’s heme oxygenase-1 (HO-1) offers antioxidant potential through heme catabolism and the production of CO, ferritin, and bilirubin [19,20,21,22]. Evidently, oxidative stress emerges as a central protagonist in an array of acquired and congenital gastrointestinal disorders, as well as a mediator of adverse effects from therapeutic interventions and procedures. An overview of the current evidence and mechanisms that implicate oxidative stress in GI disorders are summarized in Table 1 and Figure 1 and discussed below.

### 2.1. Oxidative Stress in GI Disease 

#### 2.1.1. Drug/Toxin Exposure

Chemotherapeutic agents initially produce high levels of ROS, which are a primary cause of pathology following the use of agents such as anthracyclines (daunorubicin, doxorubicin), platinum-based compounds (cisplatin, carboplatin, oxaliplatin), epipodophyllotoxins (e.g., etoposide and tiliroside), alkylating agents, and camptothecins [84]. In addition, the administration of mitoxantrone, actinomycin D, enediynes such as bleomycin, elasmin A, chartreusins, 5-fluorouracil (5-FU), and irinotecan can induce severe oxidative stress [85,86,87]. It is widely considered that chemotherapeutic agents depend largely on ROS generation to destroy cancerous cells [88]. However, ROS also contribute to many common side effects, including GI toxicity and mutagenesis [85,89]. The deleterious effects of the overproduction of ROS include severe mucosal damage [31,84], loss of epithelial cells and tight junction proteins [90], microbiota imbalance [91,92], and enteric neuropathy [30]. Nearly all chemotherapy patients suffer from GI side effects, such as nausea, vomiting, diarrhea, constipation, and ulceration [30,93]. Given their negative impact on medication adherence, these side effects are a constant challenge for patients undergoing cancer treatment [94,95].

Mucositis is one of the most undesired side effects of antineoplastic chemotherapeutics, presenting as severe inflammation of the GI mucosa [96]. Chemotherapy-induced mucositis is responsible for poor clinical outcomes, including an increased risk of infection, prolonged hospitalization, and even death [97]. Mucositis is associated with various symptoms, such as nausea, severe diarrhea, GI bleeding, and severe abdominal pain [98]. It is well established that the pathogenesis of mucositis correlates with the overwhelming production of ROS and inflammatory mediators [99]. The primary mediator of mucosal damage after chemotherapy is the overproduction of ROS [98]. Oxidative stress leads to DNA damage in epithelial progenitor cells, increased production of inflammatory mediators, cellular apoptosis, and a progressive loss of cells from the absorptive surface. The findings of Rtibi et al. [31] showed that irinotecan causes GI stress via oxidative stress-induced disturbances in water and electrolyte transport in the intestinal mucosa in rats [31]. Similarly, 5-FU, another chemotherapeutic drug known for inducing intestinal mucositis, is associated with the modulation of antioxidant defense mechanisms and stimulation of ROS generation in a mouse model [32]. Together, these studies indicate that ROS generation can initiate and promote inflammation in the intestinal mucosa. While the bulk of research has focused on the mechanisms of oxidative stress in the intestinal mucosa, ROS generation by chemotherapeutic agents has been identified as a key driver of nervous system dysfunction in the gut, which will be discussed later in this review [30,100].

Radiotherapy is an important treatment modality for abdominal and pelvic malignancies; however, GI complications and enteropathy are common sequelae after exposure. Oxidative stress is considered the driving force through which radiation induces cell death in neoplastic cells [101]. Radiotherapy generates a large number of free radicals, which are predominantly formed by the radiolysis of water to •OH but can also be produced by the mitochondria [102]. These free radicals target nuclear DNA and cell structures to induce cell death in rapidly proliferating cells, leading to inevitable off-target effects on healthy cells. In models of radiation injury, irradiation results in an increase in the lipid peroxidation product malondialdehyde (MDA), indicative of oxidative stress in the small bowel [54]. Furthermore, irradiation results in a decrease in enzymatic antioxidant defenses, including SOD and CAT, which could potentially hinder the ability of the gut to buffer against oxidative stress and cause long-term damage [54].

Nonsteroidal anti-inflammatory drugs (NSAIDs) are generally considered safe and are widely used in the clinical setting for their analgesic and anti-platelet properties. Nevertheless, there is a clear risk of developing GI complications, including gastroduodenal ulcers, with prolonged NSAID usage. The metabolites of NSAIDs can have pro-oxidant properties [103] or induce the generation of ROS in other cells. For example, indomethacin can increase mitochondrial O_2_^.−^ and XOD expression directly in colonic epithelial cells in in vitro models, resulting in increased O_2_^.−^ generation [104,105]. Oxidative stress is recognized as critical to the pathogenesis of gastroduodenal ulcers. Early evidence of this association included observations of the depletion of antioxidant enzymes, such as SOD, in gastric and duodenal biopsies [46]. Other studies demonstrate that free radicals have direct toxic effects on the GI tract. This includes observations that the free radical generator XOD causes a substantial gastric injury independent of acid secretion [47] and similar effects are caused by the administration of H_2_O_2_ [48], both of which are reversed by SOD administration. Specifically, SOD and CAT have been shown to reduce gastric injury by indomethacin in experimental models, suggesting that the harmful effects of NSAIDs are mediated by O_2_^.−^ and H_2_O_2_ [49].

Chronic alcohol consumption is also associated with GI toxicity, and chronic intestinal pseudo-obstruction has previously been linked to fetal alcohol syndrome [106]. Alcohol consumption results in oxidative stress via the oxidative byproducts of ethanol metabolism and nicotinamide adenine dinucleotide (NAD) depletion. In models of chronic alcohol exposure, protein nitration has been associated with damage to the intestinal barrier, increasing its permeability and implicating NO in its pathogenesis, notably prior to any evidence of liver disease [34]. Beyond the liver, enzymes responsible for ethanol metabolism can be found in the gut, including high levels of cytochrome P450 2E1 (CYP2E1), which generates free radicals that were shown to trigger oxidative stress-dependent changes in epithelial barrier permeability in in vitro models [35]. Further evidence of a direct oxidative stress mechanism in epithelial cells includes a decrease in tight junctions and an increase in O_2_^.−^ in response to ethanol and its metabolite acetaldehyde in the Caco-2 cell model in vitro [36]. Notably, effects on tight junctions were suppressed by the application of the antioxidant N-acetyl cysteine (NAC), suggesting that alcohol induces oxidative stress directly in epithelial cells and increases barrier permeability in the intestine.

#### 2.1.2. Ischemia–Reperfusion and Postoperative Injury

Ischemia–reperfusion injury is an important clinical problem for ischemic syndromes and solid organ transplantation and occurs in several tissues upon reoxygenation. The mechanisms involved are largely considered to be driven by oxidative stress followed by the activation of an immune response in the injured tissue [107]. The gut is considered to be highly susceptible to ischemia–reperfusion injury due to its ability to generate a large number of free radicals. Xanthine dehydrogenase and XOD are interconvertible enzymes from the same gene product [108]. During intestinal ischemia, xanthine dehydrogenase is converted to XOD, which is capable of producing the free radicals O_2_^·−^ and H_2_O_2_ from oxygen. In the ischemic environment deprived of oxygen, this is of little consequence; however, reoxygenation results in a rapid influx of the oxygen substrate, which is subsequently converted to a burst of free radicals, causing oxidative stress [43,109]. Further contributors to oxidative stress include iNOS and MPO, which are mediators of the immune response preceding cell and tissue injury [43]. Furthermore, treatment with the antioxidants NAC, SOD, or allopurinol can prevent tissue damage and inflammation in the gut caused by ischemia–reperfusion injury, highlighting the importance of O_2_^.−^ and XOD in the pathogenesis of ischemia–reperfusion injury [43,109].

Tissue ischemia resulting in bowel injury can be caused by several conditions and often requires surgical resection to remove the affected tissue. Given that ischemia–reperfusion is associated with oxidative stress, this process was modeled in rats to evaluate the effects of hypoxia by portal vein occlusion on bowel anastomotic healing. In this study, oxidative stress, as measured by lipid peroxidation and protein oxidation, was associated with poor anastomotic healing and a lack of collagen deposition [26]. Nevertheless, ischemia is not necessarily required to induce oxidative stress in bowel anastomoses. The physical bowel injury from surgery alone also appears to evoke oxidative stress in intestinal tissues. In experimental models evaluating anastomotic healing, inhibition of iNOS was found to increase the pressure required for bowel rupture, which was associated with a decline in lipid peroxidation and decreased levels of SOD, consistent with lower oxidative stress [29]. Likewise, ozone treatment has been shown to reduce anastomotic leaks, with concurrent suppression of lipid peroxidation and reduced levels of MPO [25]. The mechanism through which ozone promotes anastomotic healing remains unclear as it possesses free radical properties, but it was postulated, however, to stimulate the expression of antioxidant defense enzymes when present at low levels, the latter of which was supported by an increase in SOD and glutathione peroxidase activity [25]. Notably, the lipid peroxidation product MDA has been found to be highly predictive of anastomotic leaks in patients after elective rectal surgery when evaluated in serum and surgical drain fluid on postoperative day 3. This further suggests that oxidative stress negatively impacts wound healing and provides a novel prognostic marker that may be used in the clinical setting [27]. Ileal pouch–anal anastomosis in ulcerative colitis (UC) patients may be complicated by pouchitis, which is likely secondary to bacterial stasis. In an animal model of this procedure, oxidative stress was confirmed by increased levels of 8-isoprostane in the urine and MPO activity in the gut [41]. While this may also be associated with an inflammatory process, allopurinol (which inhibits oxidative stress) and the non-enzymatic antioxidant vitamin E prevented pouchitis, supporting a critical role for oxidative stress in the development of inflammation from bacterial stasis.

Postoperative ileus, or paralytic ileus, is a common condition in which the peristaltic activity of the intestine is diminished after bowel manipulation during surgical procedures. Many studies have investigated the mechanisms of postoperative ileus, revealing a complex condition that involves an initial activation of cells in the muscularis propria [110]. These cells may include resident macrophages, mast cells, enteric glia, and enteric neurons and potentially an influx of infiltrating immune cells, such as monocytes and neutrophils, at later stages that disrupt normal intestinal motility [110,111]. Notably, in a mouse model of postoperative ileus, oxidative stress was indicated by elevated lipid peroxidation levels as early as the first postoperative hour [78]. MPO and iNOS are two of the largest sources of immune system-derived ROS/RNS and were upregulated in this model; however, this did not occur until 3 h postoperatively and coincided with the onset of inflammatory cytokine production, including interleukin-6 (IL-6) and monocyte chemoattractant protein-1 (MCP-1) [78]. This suggests that oxidative stress may occur independent of inflammation in the immediate stage following tissue injury and instead may have a role in promoting the inflammatory response through the rapid oxidative burst. The same authors demonstrated that pretreatment with a carbon monoxide (CO) donor prevented oxidative stress and postoperative ileus, which was at least partially dependent on the activation of the antioxidant enzyme HO-1. Interestingly, transient receptor potential melastatin 2 (TRPM2) was found to be expressed by muscularis macrophages, and its deletion completely negated postoperative ileus in a murine model [112]. As TRPM2 is known to be an important ROS sensor, this could provide a mechanistic link through which an initial elevation in ROS could shape the local immune response in the muscularis propria of the intestine.

#### 2.1.3. Congenital Disorders

Genetic GI conditions may also be primarily associated with oxidative stress, though due to the complexity of mutations, more research in this space is required. One example is Triple-A syndrome, whereby esophageal achalasia presents as a key feature. In this disease, mutation of the *AAAS* gene likely results in disturbed redox homeostasis, as demonstrated by oxidized and reduced glutathione balances after the knockdown of *AAAS* in human adrenocortical tumor and neuroblastoma cells [57]. Subsequently, these cells are highly susceptible to cell death following oxidative insult [57]. Other conditions associated with oxidative stress could include forms of chronic intestinal pseudo-obstruction that are associated with mutations in mitochondrial genes and mitochondrial neurogastrointestinal encephalopathy (MNGIE). Given that the mitochondria are the largest source of intracellular ROS, damage to the mitochondria could cause the uncoupling of oxidative phosphorylation and hampering of detoxifying mechanisms, which could contribute to the underlying pathophysiology of these diseases [113]. Infantile hypertrophic pyloric stenosis is another multifactorial genetic disease that can cause debilitating obstruction. While the etiology of this condition is unclear, it has been associated with a lack of NO production and can be modeled in the *Hph-1* knock-out mouse model, which lacks tetrahydrobiopterin (BH4), an important cofactor for NO synthesis [80]. In this model, nNOS was upregulated as a possible compensatory mechanism; however, elevated levels of H_2_O_2_ and O_2_^.−^ are suggestive of possible nNOS uncoupling [80]. A recent report has also implicated oxidative stress as a possible contributing factor to Hirschsprung disease, a multifactorial genetic disease whereby neural crest-derived cells fail to migrate within the gut wall during GI tract formation. In this model, pups exposed to intrauterine oxidative stress failed to form an enteric nervous system, suggesting that this may be one of the many predicted environmental factors that contribute to disease penetrance [83]. Interestingly, the NSAID ibuprofen has also been associated with inhibition of bowel colonization by neural crest-derived cells through a COX-independent mechanism, though oxidative stress was not evaluated [114].

#### 2.1.4. Inflammation and Infection 

Oxidative stress constitutes an essential part of normal immune system functions, and free radicals are utilized as a delicate tool to eliminate bacteria and unhealthy cells and to modify the cellular response by inducing inflammatory transcriptional pathways and altering protein function. Nevertheless, free radical signaling acts in a non-specific manner, which can result in unwanted tissue damage reciprocally driving inflammatory processes. This has been best studied in inflammatory bowel disease (IBD) within GI research. IBD collectively encompasses UC and Crohn’s disease (CD), two incurable conditions in which patients experience periods of remission, flares, and relapses of inflammation within the GI tract that ultimately requires bowel resection in up to 80% of cases [115,116,117]. In UC, inflammation is observed in the colon and rectum, whilst in CD, the entire GI tract, but predominantly the colon and terminal ileum, may be inflamed [115,116]. Oxidative stress is prominent in IBD patients and experimental models of colitis [73,75,118,119]. A decline in the scavenging of free radicals is reported in IBD patients [71,73]. Moreover, markers of severe oxidative stress are evident in both UC and CD patients [70,74]. In colitis, the activity of several enzymes that participate in the inflammatory response and the endogenous production of ROS can be increased, including NOX, NOS, LOX, COX, and MPO [72,75]. Oxidative stress acts as an important inflammatory amplifier as ROS can directly upregulate several genes involved in the inflammatory response and increase mucosal permeability, resulting in enterotoxic and antigenic insult, which perpetuates intestinal inflammation [120]. Notably, there is also evidence that oxidative stress can precede the onset of the immune response and may contribute to the pathogenesis of chronic intestinal inflammation [121].

Necrotizing enterocolitis (NEC) is a life-threatening GI disease affecting premature and low birthweight infants. NEC also has important links to tissue oxygenation levels in the pathogenesis of the disease. This condition is considered multifactorial and involves an element of immune stimulation in the premature intestine during colonization with intestinal flora [122]. Total oxidant status (TOS) and the oxidative stress index (OSI) are increased in the serum of patients with NEC compared to preterm healthy controls, which further correlate with disease severity [59]. Notably, markers of oxidative stress, including advanced oxidation protein products and total hydroperoxides, in cord blood are predictive of developing NEC, which suggests that disturbances in the redox balance may precede disease onset [60]. After birth, there is a dramatic shift in oxygenation from the intra to the extrauterine environment. At later stages of gestation, antioxidant defenses are strengthened prior to birth [58]. Given that 90% of NEC cases occur in preterm infants, prevailing hypotheses surrounding oxidative stress in NEC include an inability to successfully modulate free radical scavenging, which can be accentuated by O_2_ supplementation, and formula feeding, which lacks the antioxidant properties of breast milk. Notwithstanding, inflammation is a critical aspect in the pathophysiology of NEC with the pattern recognition receptor toll-like receptor 4 (TLR4) implicated as a key driver of the disease [122]. Notably, lipopolysaccharide (LPS), the bacterial ligand for TLR4, has been shown to increase O_2_^.−^ production via NOS uncoupling in rat models of NEC, which implicates oxidative stress in mediating later stages of the disease [61]. 

Oxidative stress has also been implicated in parasitic infections of the intestine. Chagas disease is caused by the *Trypanosoma cruzi* (*T. cruzi*) parasite infection, but megacolon or megaesophagus occurs only in a subset of patients. Recently, the *MRPS18B* P260A gene variant was identified in 38.4% of patients with Chagas megaesophagus compared to 2.2% of the asymptomatic population [64]. To ascertain the potential function of the gene variant, the authors generated the Epstein–Barr virus-immortalized B lymphoblastoid cell lines from patients and showed that stimulation of these cells with the cytokine interferon-γ resulted in increased protein nitration and O_2_^.−^ generation from the mitochondria, implicating nitro-oxidative stress as a potential mechanism. Interestingly, though oxidative stress is often utilized by immune cells to eliminate pathogens, *T. cruzi* proliferation is regulated by the redox status of its environment, with a pro-oxidative redox state enhancing its proliferation [123]. Subsequently, the application of the antioxidant NAC promoted the parasitic burden in the midgut of its insect vector *R. prolixus* [123]; however, the implications for this in human disease are not yet clear. 

#### 2.1.5. Cancer

It is established that intestinal inflammation causes oxidative stress which results in a high burden of free radicals secondary to the pathology, but inflammation also greatly enhances the risk of carcinogenesis, and oxidative stress may be considered a key instigator. In IBD, there is a substantial increase in the risk of developing colorectal cancer. Additionally, there is an increased risk of other malignancies, including small bowel cancer and extra-intestinal cancers, all of which have been linked to chronic oxidative stress-induced mutagenesis [124,125,126]. Barrett’s esophagus similarly involves oxidative stress, ultimately contributing to the development of esophageal adenocarcinoma [127]. In Barrett’s esophagus, the squamous mucosa of the distal esophagus is replaced by metaplastic columnar epithelium after prolonged insult, predominantly by the reflux of gastric acid and bile salts in gastroesophageal reflux disease. In biopsies of Barrett’s esophagus, there is an increase in levels of peroxynitrite, O_2_^.−^, and GSH, indicating oxidative stress [23]. Additionally, levels of the antioxidant enzymes CuZn-SOD, Mn-SOD, and CAT are elevated, which is suggestive of an adaptive response to elevated oxidative stress. Nonetheless, SOD activity was shown to be decreased [23], and progression to adenocarcinoma has been associated with the silencing of glutathione peroxidase and glutathione S-transferases, which have antioxidant properties [128,129]. While inflammation is involved, free radicals could be produced directly by the epithelium as elevated levels of NOX5 are observed in biopsies of Barrett’s esophagus and esophageal adenocarcinoma, and pulsed-acid treatment resulted in increased H_2_O_2,_ which could be inhibited by blocking NOX, thereby providing a mechanism through which reflux can directly induce oxidative stress [24]. 

While oxidative stress mediates the therapeutic effects of radiotherapy and chemotherapy in the treatment of cancer, oxidative stress is also a major contributor to carcinogenesis itself. In colorectal cancer, free radicals derived from environmental sources, diet, sedentary lifestyle, and inflammation can lead to oxidative stress and cause genomic instability and mutagenesis, resulting in the transformation of healthy colonocytes to dysplastic and neoplastic cells [126]. Biopsies of primary colorectal tumors support oxidative stress-dependent mechanisms of genomic instability, given the presence of oxidized DNA adducts and increased lipid peroxidation products, MDA and 4-hydroxynonenal (4-HNE), both of which positively correlate with histological grade and clinical stage [38]. Notably, neoplastic cells appear to adapt to the pro-oxidative environment, with reports of upregulation in CuZn-SOD, glutathione peroxidase, and GSR, which may promote tumor survival, while conversely, non-enzymatic antioxidants, including vitamins C and E and reduced glutathione, can be decreased [38]. 

#### 2.1.6. Diabetes Mellitus

A large body of research exists regarding the role of oxidative stress in tissue injury from diabetes mellitus. Oxidative stress may be largely considered a secondary driver of the disease pathophysiology as the primary effects appear to be mediated by advanced glycation end product (AGE) signaling and low-grade inflammation. In the GI tract, diabetes is associated with the development of gastroparesis and intestinal dysmotility. In the non-obese diabetic mouse model of gastroparesis, only a portion of mice develop delayed gastric emptying [19,20]. In the mice that exhibit this phenotype, elevated lipid peroxidation (as indicated by MDA levels) is observed compared to those with normal gastric emptying. Loss of nNOS (nitrergic neurons) and c-KIT (interstitial cells of Cajal, ICC) is observed, which normally mediate the relaxation of the pyloric sphincter to allow transit of gastric contents. Interestingly, the mice that do not develop delayed gastric emptying exhibit higher expression of the antioxidant enzyme HO-1, which was later shown to be expressed by gastric macrophages and may serve to protect cells of the muscularis propria from oxidative stress [19,20]. Oxidative stress has also been implicated in the streptozotocin (STZ) model of diabetes, with reports of elevated lipid peroxidation and protein oxidation after 6 weeks [66]. Other studies place the effects of STZ as more immediate with elevated O_2_^.−^, depletion of GSH, and increased CAT activity observed in the duodenum after only 5 days [67]. These effects could be reversed by insulin, validating that these observations were a direct result of hyperglycemia. Finally, recent studies have found that the consumption of ultra-processed foods, which have been associated with an elevated risk of developing type 2 diabetes, promote ROS production to induce a systemic pro-oxidant and proinflammatory state, suggesting another mechanism through which oxidative stress may drive disease pathogenesis [130].

## 3. Impact of Oxidative Stress on the Enteric Nervous System and Associated Sequelae

The ENS, an integral component of the autonomic nervous system, intricately regulates the physiological functions of the GI tract by orchestrating effector systems like musculature, secretion, and vasculature. This distinctive influence has positioned the ENS as an alluring and innovative therapeutic target for a spectrum of GI disorders [93,131]. Structurally, this intricate neural network comprises two distinct plexuses—the outer myenteric plexus and the inner submucosal plexus [132]. The myenteric plexus is characterized by its location between the outer longitudinal and inner circular smooth muscle layers of the intestinal wall, where enteric neurons control the rhythm and coordination of muscular contractions and engage with tissue-resident muscularis macrophages [133]. Conversely, the submucosal plexus is situated within the interstice between the muscular and epithelial layers, steering the nuanced control of mucosal functions such as permeability, secretion, absorption, leukocyte migration, and the dynamic regulation of blood flow [133,134].

Dysfunction of the ENS is a hallmark of several gastrointestinal disorders, encompassing alterations in its structure, neuroinflammatory responses, aberrations in neuronal excitability and signaling, and conditions such as enteric neuropathy or aganglionosis. This disruption leads to imbalances in gut homeostasis, often culminating in dysmotility or aperistalsis. This constitutes a spectrum of disorders comprised of idiopathic gastroparesis, Hirschsprung’s disease, esophageal achalasia, Chagas disease, pyloric stenosis, mitochondrial neurogastrointestinal encephalomyopathy (MNGIE), and certain manifestations of chronic intestinal pseudo-obstruction, collectively termed as neurointestinal diseases, which underscore the profound impact of ENS aberrations on gut physiology [135]. Conditions such as IBD, diabetic gastroparesis, and chemotherapy-induced GI complications, even though triggered by different pathogenesis, exhibit comparable disruptions in neurally mediated functions, contributing to significant morbidity. Interestingly, preclinical studies in models of Parkinson’s disease have demonstrated enteric neuroinflammation in the early stages of disease pathogenesis prior to the onset of neurological symptoms, suggesting that the deleterious effects of enteric oxidative stress may also be pertinent as a biomarker or a part of the pathophysiology of disorders affecting the central nervous system (CNS) [136,137]. The consequences of ENS aberrations are far-reaching, inducing a spectrum of debilitating sequelae ranging from profound dysmotility to potentially fatal complications like impaction or perforation. The restoration of ENS integrity emerges as a paramount objective not only to enhance quality of life but also to avert these grave complications. Intriguingly, a common thread among several acquired GI disorders involving ENS dysfunction is their association with oxidative stress. This intricately woven relationship between neuronal damage and oxidative stress remains an active area of investigation, seeking to illuminate the mechanistic underpinnings and potential therapeutic avenues to mitigate these debilitating conditions (Table 1).

### 3.1. Antioxidant Defense in Enteric Neurons 

In general, neurons are particularly susceptible to oxidative insult from free radicals due to their higher energy demand and O_2_ consumption, excess mitochondria-derived O_2_^.−^, auto-oxidation of neurotransmitters, excitotoxicity, poor antioxidant defenses, and limited replicative potential [138]. Neurons of the ENS are also highly sensitive to oxidative stress, which has been shown to alter their electrophysiological properties, damage neuronal membranes, and trigger neuronal death [69,139,140]. The role of oxidative stress in neuronal damage was shown in models of chemotherapy, diabetes, physiological aging, and Chagas disease [30,53,65,68,141]. Similarly, oxidative stress is predicted to be a key contributor to ENS dysfunction in the pathophysiology of IBD [118,131]. In physiological aging of the intestine, enteric neuron loss was observed in mice after 17 months and associated with increased ROS and markers of apoptosis [53]. Notably, this can be prevented by calorie restriction, which is an established method to limit cumulative damage by ROS through metabolic pathways. The same study found that the potent ROS inducer menadione enhances enteric neuropathy; however, this effect was not observed in younger mice at 6 months of age, suggesting that neurons become less adept at buffering oxidative stress at later stages of life [53]. In diabetic enteric neuropathy of the colon, the implications of oxidative stress were studied in enteric ganglia isolated by laser capture microdissection. Specifically, there was a lower expression of reduced glutathione within the enteric ganglia, indicating local redox imbalance in the diabetic condition [68]. However, SOD was concurrently elevated in enteric ganglia in this model, which may indicate that the ENS can compensate for oxidative insult to some degree. Another important contributor to the antioxidant defense of enteric neurons is GSH, which protects enteric neurons from H_2_O_2_-induced cell death in ex vivo intestinal tissues, and therefore appears to be an important mediator in preventing oxidative stress-induced injury [142]. In the first and rate-limiting step, glutamate–cysteine ligase (GCL) catalyzes the formation of γ-glutamylcysteine (γ-GC) from glutamate and cysteine. γ-GC then combines with glycine in a reaction catalyzed by glutathione synthetase (GS) to form reduced GSH (γ-glutamyl-cysteinyl-glycine). GCL is predominately expressed by enteric glia, while neurons have high levels of GS, indicating that enteric glia may provide the substrate for GSH production by neurons similar to what occurs in the CNS [143]. Notably, the blockade of GCL by l-buthionine-sulfoximine (BSO) resulted in enteric neuropathy in ex vivo preparations of the colon [143]. Furthermore, the silencing of GCL specifically in enteric glial cells using viral delivery of shRNA negated their neuroprotective ability in an in vitro model of dopamine toxicity associated with oxidative stress [142]. This suggests that cells surrounding enteric neurons are equally important to their resistance to oxidative injury. This could also include other cells in the region, such as muscularis macrophages that express HO-1 [20]. 

### 3.2. Mechanism of Enteric Neuropathy Involving Oxidative Stress 

**Figure 2:**
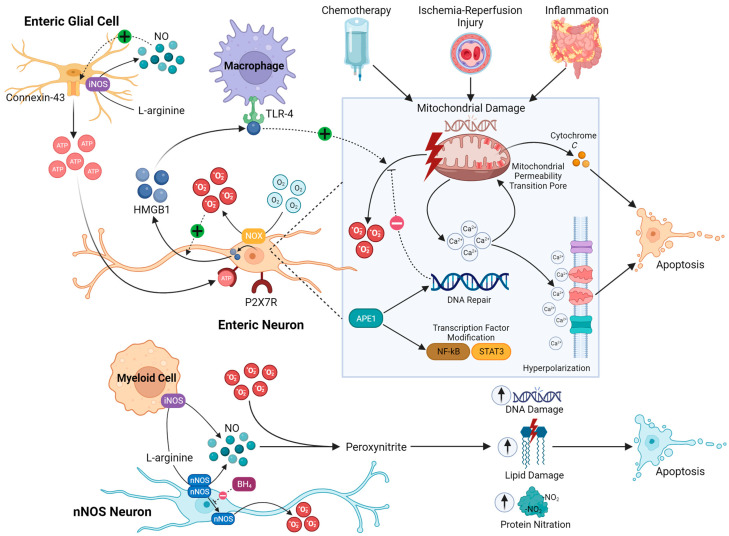
Mechanisms through which oxidative stress induces enteric neuropathy. In the intrinsic nitrosative injury pathway, NO produced by nNOS enteric neurons can react with O_2_^.−^ to form peroxynitrite. Peroxynitrite subsequently damages DNA, lipids, and proteins, resulting in either dysfunction or destruction of nNOS neurons. Additionally, nNOS produces O_2_^.−^ in its monomeric form (while dimeric nNOS produces NO), which is favored in the absence of BH4 (which serves to stabilize nNOS dimers). Beyond intrinsic NO production by enteric neurons, nitrosative injury may also be mediated by excess NO production by glia and immune cells expressing inducible NOS (iNOS). NO has also been shown to stimulate ATP release from enteric glia via connexin-43 channels, which then binds P2X7R on enteric neurons, resulting in neuronal death. A second pathway through which oxidative stress causes neuropathy involves enteric neuron mitochondrial dysfunction. Enteric neurons have been found to express the enzyme NOX, which converts O_2_ to O_2_^.−^ and may have increased activity under hyperoxic conditions (as seen in the CNS and peripheral nervous system (PNS)). O_2_^.−^ may then feed into other oxidative stress pathways, such as the intrinsic nitrosative pathway. Various oxidative insults (including chemotherapy, ischemia–reperfusion injury, and inflammation) trigger mitochondrial and mitochondrial DNA damage, leading to dysfunction of the electron transport chain and increased cytosolic calcium levels. This cytosolic calcium triggers sustained hyperpolarization of the neuron cell membrane and the release of cytochrome *c* from mitochondria via the mitochondrial permeability transition pore, both of which promote enteric neuronal death. Thirdly, oxidative stress from intestinal inflammation can drive enteric neuropathy. High levels of O_2_^.−^ in inflammation promote the release of HMGB1 from enteric neurons, which binds and activates TLR4 on immune cells, stimulating further mitochondrial O_2_^.−^ production in enteric neurons and likely representing a positive feedback loop. Myenteric ganglia also express apurinic/apyrimidinic endonuclease 1/reduction–oxidation (redox) effector factor-1 (APE1/Ref-1), which participates in both DNA repair and modification of proinflammatory transcription factors (including NFκB and STAT3), the latter of which is enhanced under oxidative conditions. Inhibition of APE1/Ref-1′s redox functions and activation of its DNA repair function was shown to attenuate mitochondrial O_2_^.−^ production, DNA damage, and cell death in myenteric neurons.

Currently, the known mechanisms of oxidative stress in enteric neurons can be categorized as related to either intrinsic nitrosative injury, mitochondrial dysfunction, or inflammation-related oxidative stress (Figure 2). However, these pathways may not always be mutually exclusive. Intrinsic nitrosative injury can often be identified by excessive loss of nNOS neurons; however, in intestinal inflammation and chemotherapy exposure, nNOS-expressing neurons are not specifically lost [119,144,145,146,147,148,149,150,151]; therefore, non-nitrosative stress mechanisms of enteric neuropathy are more likely in these conditions.

#### 3.2.1. Intrinsic Nitrosative Injury 

Intrinsic nitrosative injury in enteric neurons relates to their endogenous ability to produce NO through nNOS, which acts as a critical inhibitory neurotransmitter within the ENS. In disease conditions, the nitrosative product NO is also considered to contribute to oxidative stress. O_2_^.−^ and NO can react to form the compound peroxynitrite, which damages proteins, lipids, and DNA [15]. The lack of protection of the ENS exposes enteric neurons to oxidative stress, eliciting neuropathy, neuronal degeneration, and altered neuronal functions resulting in intestinal dysmotility [68]. It has been demonstrated in vivo that oxidative stress in intestinal ischemia–reperfusion injury, diabetes, and chronic alcohol consumption is associated with a selective loss of nNOS-immunoreactive enteric neurons, which may be explained by a nitrosative mechanism of damage [44,45,152,153,154]. Similar observations are made when oxidative stress is induced artificially in enteric neurons in vitro using menadione sodium bisulfite [155]. In a model of intestinal ischemia–reperfusion injury, protein nitrosylation and apoptosis of nNOS neurons were observed as early as 6 h post-injury, indicating a rapidly acting mechanism of enteric neuropathy [45]. In Chagas disease, enteric neuropathy was associated with protein tyrosine nitration in enteric neurons. In vitro studies revealed that infection of enteric neurons with *T. cruzi* directly leads to elevated production of NO, nitrotyrosine, and mitochondrial membrane depolarization [65]. While the intrinsic nitrosative injury is considered a consequence of the disproportionate propensity of nNOS neuron loss, similar nitrosative mechanisms in enteric neuropathy could be caused by excessive NO derived from iNOS in glia or immune cells, which could potentially drive indiscriminate neuronal loss [69]. NO could also drive enteric neuropathy by promoting ATP release from connexin-43 channels in enteric glia that may cause enteric neuronal death by the activation of P2X7R [145]. It is also worth mentioning that the functional production of neuronal NO is dependent on nNOS dimerization. Notably, a lack of nNOS in its dimerized form is observed in mouse models of diabetic gastroparesis, which was associated with reduced levels of BH4, a necessary cofactor that stabilizes nNOS dimers to promote NO synthesis [153]. Further, BH4-deficient mice acquire hypertrophic pyloric stenosis and have uncoupled nNOS as well as elevated O_2_^.−^ and H_2_O_2_ [80]. Therefore, it is possible that nNOS-associated enteric neuropathy could be mediated by O_2_^.−^ rather than NO.

#### 3.2.2. Mitochondrial Dysfunction 

Myenteric neurons appear to exhibit the highest density of mitochondria, which may explain their susceptibility to increases in O_2_^.−^ production in pathological conditions [30,156]. Local oxygen gradients could also affect ROS generation by the mitochondria and the redox balance. Excessive ROS are produced in a lower-energy oxidative environment or a higher-energy reductive environment [85,157]. A reductive environment is usually attributed to hypoxic conditions, and tissue hypoxia has been studied in depth in the brain as a cause of neuronal loss mediated by ROS released by damaged mitochondria [158]. Under physiological conditions, the kinetics of O_2_^.−^ production by the mitochondria are directly proportional to the levels of O_2_; this has been demonstrated in neurons of the peripheral nervous system (PNS) and CNS [159,160,161,162]. Therefore, there is also potential for a hyperoxic environment to cause neuronal damage, as shown in cortical neurons [163]. Notably, enteric neurons have been reported to express O_2_^.−^-generating NOX enzymes; however, the physiological implications of this are currently unclear [164]. Mitochondria are also critical to maintaining the electrophysiological properties of enteric neurons. Inhibiting complexes of the electron transport chain causes mitochondrial dysfunction and increases cytosolic Ca^2+^ levels, which results in sustained hyperpolarization [156]. Neurons are dependent on the mitochondria, Ca^2+^ signaling, and ion transport for homeostatic signaling; oxidative stress can cause dysfunction in all these processes, thus providing a potential explanation for their perturbed function under oxidative conditions [165,166,167]. Likewise, a high Ca^2+^ load is associated with increased mitochondria-derived ROS and can contribute to cell death through the voltage- and Ca^2+^-dependent mitochondrial permeability transition pore [165]. The codependency of ROS and Ca^2+^ transport in enteric neurons may explain their propensity for oxidative stress-induced injury.

Mitochondrial dysfunction has been strongly linked to enteric neuropathy caused by chemotherapy. Treatment with the platinum-based chemotherapeutic drug oxaliplatin induces the loss of enteric neurons, including nNOS-expressing neurons in the submucosal and myenteric plexuses. This correlated with severe colonic dysmotility and constipation [30]. The proportion of nNOS-immunoreactive neurons after oxaliplatin exposure was higher in the submucosal and myenteric plexuses of the distal colon, indicating that this is likely not the result of an intrinsic nitrergic neuropathy as observed in other GI conditions. Nevertheless, an overabundance of NO appeared to be critical to the pathology of dysmotility, with an increased amplitude of NO-mediated slow inhibitory junction potentials in the smooth muscle and NO-dependent reduction in the frequency of colonic migrating motor complexes. Furthermore, increased nitrotyrosine was observed in both submucosal and myenteric neurons despite a non-specific loss of nNOS-immunoreactive neurons. The expression of iNOS was elevated in the longitudinal muscle–myenteric plexus preparations after oxaliplatin treatment and, therefore, may have contributed to RNS formation in neurons, leading to dysmotility. In the submucosal and myenteric ganglia, an increase in O_2_^.−^ derived specifically from the mitochondria was observed with concomitant enhancement in mitochondrial membrane permeability, a critical step in cell death via the release of cytochrome *c* and a likely mediator of enteric neuropathy in this condition [30]. Notably, the pharmacological compound BGP-15 was found to reverse oxidative stress in enteric neurons and restore motility [141]. This compound exhibits promiscuous, but complementary, mechanisms to prevent cell death including inhibition of poly-ADP ribose polymerase (PARP), co-induction of heat shock protein (HSP)72, and antioxidant activity. Therefore, it is not completely clear whether mitochondrial O_2_^.−^ production was alleviated directly by this compound or downstream of other neuroprotective processes [141].

Other conditions that may involve enteric neuropathies that could be caused by mitochondrial dysfunction in neurons include various genetic diseases. Enteric neuropathies are readily observed in the forms of chronic intestinal pseudo-obstruction with mutations in genes with mitochondria-specific functions. Notably, MNGIE, a genetic condition leading to cumulative mitochondrial DNA damage, primarily manifests with severe GI dysmotility and leads to enteric neuropathy and smooth muscle atrophy, highlighting the vulnerability and significance of mitochondrial damage in the muscularis propria region of the intestine [168]. Recently, biallelic variants in *LIG3* were associated with enteric neuropathy and were identified to cause mitochondrial dysfunction, including excess O_2_^.−^ production, in MNGIE patient-derived fibroblasts [81].

#### 3.2.3. Enteric Neuroinflammation

Oxidative stress and chronic neuroinflammation intertwine as key pathologic factors contributing to enteric neuropathy [131,149]. This is illustrated in chemically-induced colitis, which causes oxidative stress in the ENS and consequently dysfunction in neurally controlled intestinal functions [140]. In a model of parasitic ileitis, the greatest changes in lipid peroxidation were observed in the muscle layers rather than in the mucosa or plasma [169]. Therefore, the muscle layers of the intestine appear to be susceptible to oxidative injury. Likewise, myenteric neurons contained within the muscle layers are not resistant to oxidative stress induced by intestinal inflammation [69]. In intestinal inflammation, the role of oxidative stress in mediating neuronal cell death is exemplified by the administration of the antioxidant NAC, which attenuates neuronal loss in vivo in an acute model of dinitrobenzene sulfonic acid (DNBS)-induced colitis [69]. Additionally, NAC did not appear to directly ameliorate the inflammatory response, suggesting a greater contribution from oxidative insult than proinflammatory cytokines in driving neuropathy in colitis. In the same study, high levels of GSSG/GSH were observed, indicating that myenteric neurons were under an oxidative redox environment. Furthermore, high levels of the free radical O_2_^.−^ were observed in the myenteric plexus in the colon of mice with DNBS-induced colitis. In a genetic model of spontaneous chronic colitis closely representing UC, indiscriminate neuropathy has been observed, which was associated with high levels of O_2_^.−^ derived from the mitochondria, oxidized DNA adducts, and translocation/release of HMGB1 specifically from enteric neurons [76]. H_2_O_2_ is another free radical prevalent in the muscle layers of the colon in chemically-induced models of colitis [170]. Oxidative stress in enteric neurons is commonly modeled by applying this compound to enteric neuronal cell lines, primary cultures, and organotypic preparations, which highlights its potential role in evoking oxidative stress-induced enteric neuropathy in vivo [142,148,171,172,173,174]. HMGB1 translocation from enteric neurons was replicated when ex vivo preparations of healthy muscularis were subjected to H_2_O_2_ or hyperoxia (95% O_2_), suggesting this is a redox-sensitive process [76]. In this study, inhibition of HMGB1 by glycyrrhizic acid was found to reverse neuropathy, but not inflammation, implicating HMGB1 in driving enteric neuropathy. HMGB1 is a potent damage-associated molecular pattern (DAMP) and an endogenous ligand for TLR4 [175]; therefore, it could be a mediator of neuroinflammation and plexitis associated with the severity of IBD. Recently, other TLR4 agonists (palmitic acid and LPS) were found to elevate mitochondrial O_2_^.−^ production in enteric neurons in vitro [176]. This raises the prospect of a positive feedback loop between redox stress and HMGB1, which drives local enteric neuropathy and inflammation. Further studies identified the expression of apurinic/apyrimidinic endonuclease 1/redox effector factor-1 (APE1/Ref-1) in enteric neurons [119]. APE1/Ref-1 possesses two domains: one has a critical DNA base excision repair function, and the other modifies several transcription factors via a redox-dependent mechanism to enhance their ability to bind DNA and exert their functions [177]. This includes transcription factors involved in inflammation, such as STAT3 and NFκB. The novel compound APX3330 inhibits the redox function of APE1/Ref-1 and enhances its ability to repair DNA [119]. APX3330 treatment of mice with spontaneous chronic colitis reduced oxidative DNA damage in myenteric neurons and prevented neuropathy. Notably, APX3330 also decreased mitochondrial O_2_^.−^ production in enteric neurons, which suggests that mitochondrial dysfunction in enteric neurons probably occurs downstream of the inflammatory insult. While the majority of animal models and some human studies of IBD specimens show evidence of enteric neuropathy [131], there are several reports of hyperganglionosis in IBD specimens and evidence of neurogenesis in response to colitis to repopulate damaged neurons [178,179]. Whether the redox environment also plays a role in the regeneration of the ENS is unknown; however, a pro-oxidative environment is known to inhibit neurogenesis in the brain [180]. 

Together, these data provide compelling evidence that oxidative stress is a critical player in GI disease, and subsequent damage to the ENS is likely a major cause of dysmotility that complicates and worsens disease course. This data suggests a novel therapeutic target for the management of multiple GI conditions; however, the clinical application of antioxidant compounds has been met with limited success, predominantly due to the inadequate scavenging properties of small molecule antioxidants and low bioavailability [3]. 

## 4. Concluding Remarks 

The presented studies provide clear evidence of the contribution of oxidative stress to the pathophysiology of GI disease or corresponding sequelae via various mechanisms, including inflammation, infection, stasis, physical injury, metabolism, toxicity, and possibly genetic disease. Intriguingly, the majority of these diseases present with comorbid enteric neuropathies, changes in neuronal functions, and/or neurochemical coding, which may result in GI dysfunction. Evidence that oxidative stress contributes to dysfunction, neuropathy, or morphological changes in the ENS has been reported in IBD, diabetes, aging, ischemia–reperfusion injury, and Chagas disease; however, this phenomenon may not be limited to these conditions. The current literature suggests various mechanisms of oxidative stress-induced enteric neuropathy, including via nitrosative injury, mitochondrial dysfunction, or free radical generation associated with inflammation, which may represent novel targets for future precision therapeutics.

## Figures and Tables

**Table 1:** GI conditions associated with oxidative stress and effects on the ENS.

Condition	ENS Involvement	Evidence of Oxidative Stress Mechanisms	Citations
**Barrett’s** **esophagus/** **esophageal** **adenocarcinoma**	Unknown	In esophageal biopsies: ↑Peroxynitrite, superoxide, and GSH ↑CuZn-SOD, Mn-SOD, and CAT ↓SOD activity ↑NOX5 Pulsed acid in Barrett’s esophagus cells ↑H_2_O_2_ in a NOX-dependent manner	[23,24]
**Bowel anastomosis**	Neuropathy	iNOS inhibition increases anastomotic wound healing, ↓lipid peroxidation, ↓SOD ↑Oxidative stress induced by ischemia–reperfusion suppresses wound healing Ozone treatment ↓anastomotic leakage associated with ↓lipid peroxidation, ↓MPO, and ↑ antioxidant defenses SOD and glutathione peroxidase activity Lipid peroxidation (MDA) highly predictive of anastomotic leakage 3 days post-op	[25,26,27,28,29]
**Chemotherapeutic drugs**	Neuropathy	Irinotecan causes oxidative stress-induced disturbances in water and electrolyte transport in the intestinal mucosa 5-FU-induced intestinal mucositis associated with the modulation of antioxidant defense mechanisms and stimulation of ROS generation Oxaliplatin-induced enteric neuropathy associates with ↑nitrotyrosine and ↑mitochondrial O_2_^.−^, ↑iNOS in the muscularis propria	[30,31,32]
**Chronic alcohol** **consumption and** **fetal alcohol** **syndrome**	Decreased nNOS	Oxidative stress caused by oxidative byproducts of ethanol metabolism and NAD depletion Protein nitration and oxidation associates with intestinal barrier leakage and precedes liver disease Ethanol metabolism by intestinal CYP2E1 causes oxidative stress- dependent epithelial barrier permeability in vitro Ethanol and acetaldehyde ↓tight junctions, ↑ROS and superoxide in Caco-2 cells in vitro—reversed by antioxidant NAC Chronic ethanol exposure ↓nNOS neurons	[33,34,35,36]
**Colorectal cancer**	Neuropathy	Oxidative stress derivatives cause genomic instability and mutagenesis leading to cancer ↑Oxidized DNA (8-oxodG) ↑Lipid peroxidation products MDA and 4-HNE ↑CuZn-SOD, GSH-Px, and GSSG-R ↓Vitamins C and E and reduced glutathione	[37,38,39,40]
**Ileal pouch–anal anastomosis**	Neuropathy	↑Oxidative stress: MPO and 8-isoprostane Antioxidants allopurinol and vitamin E reduced pouchitis	[41,42]
**Ischemia–reperfusion injury**	Neuropathy	↑Oxidative stress and ROS Ischemia ↑xanthine oxidase which produces O_2_^.−^and H_2_O_2_ upon reoxygenation. Further contributions by NOS and MPO Antioxidants NAC, SOD, and allopurinol prevent tissue injury and inflammation Protein nitration and apoptosis of nNOS neurons 6h post-injury	[43,44,45]
**Gastroduodenal** **ulcers (NSAIDS)**	Changes to neurochemical coding in response to indomethacin and aspirin.	↓SOD in duodenal ulcers Free radicals can directly cause gastric mucosal injury demonstrated by administration of XOD and H_2_O_2_. Effects reversed by SOD SOD, CAT, and PGE2 reduce gastric injury to indomethacin. Allopurinol (xanthine oxidase) has no effect	[46,47,48,49,50,51]
**Physiological Aging (constipation and** **fecal incontinence)**	Neuropathy	Enteric neuron loss in aged mice 17–18 months. Increased ROS and apoptosis markers in enteric neurons of aged mice Prevented by calorie restriction ROS inducer menadione increases neuropathy but not in young mice (6 months)	[52,53]
**Radiotherapy**	Possible neuropathy	↑Lipid peroxidation product MDA in small bowel ↓CAT and SOD in small bowel	[54,55]
**Triple-A sydrome—** **esophageal achalasia**	Not shown	Deletion in antioxidant gene In vitro data demonstrated mechanism likely caused by oxidative stress due to disturbed redox balance Associated with peripheral neuropathy and dysautonomic symptoms (constipation and diarrhea)	[56,57]
**Necrotizing** **enterocolitis (NEC)**	Neuropathy	Decreased antioxidant defenses in preterm infants Oxidative stress prominent in NEC compared to preterm controls Markers of oxidative stress (advanced oxidation protein products and total hydroperoxides) in cord blood are predictive of NEC LPS-dependent superoxide production via NOS uncoupling may also mediate later stages of disease	[58,59,60,61,62,63]
**Chagas disease**	Neuropathy	Chagasic megaesophagus associated with mutation in MRPS18B P260A which causes nitro-oxidative stress Enteric neuropathy associated with protein tyrosine nitration in enteric neurons	[64,65]
**Diabetic gastroparesis and intestinal dysmotility**	Neuropathy	Non-obese diabetic (NOD) model: ↑Lipid peroxidation in diabetic mice with delayed gastric emptying ↑HO-1 in mice that do not develop delayed gastric emptying ↓ nNOS and c-kit (ICCs) HO-1 expressed by CD206+ macrophages Streptozotocin (STZ) model: ↑Lipid peroxidation and protein oxidation in small bowel after 6 weeks ↓GSH depletion, ↑O_2_^.−^ and ↑CAT activity after 5 days of induction in duodenum—STZ model Enteric neuropathy in colon ↓Reduced glutathione and increased SOD in enteric ganglia	[19,20,66,67,68]
**Inflammatory bowel disease**	Neuropathy and hyperplasia	↓Scavenging of free radicals is reported in IBD patients ↑Markers of severe oxidative stress in UC and CD patients ↑NOX, NOS, LOX, COX, and MPO Acute colitis model: Enteric neuropathy reversed by antioxidant NAC Chronic colitis model: ↑mitochondrial O_2_^.−^, oxidized DNA adducts, and translocation/release of HMGB1 from enteric neurons	[69,70,71,72,73,74,75,76]
**Postoperative ileus**	Decreased nNOS	Lipid peroxidation 1 h post-op Occurs before immune cell infiltration MPO (6 h) and iNOS (3 h) expression Occurs before proinflammatory cytokines tested (IL-6, MCP1)	[77,78]
**Hypertrophic** **pyloric stenosis**	Neuropathy	nNOS uncoupling and elevated H_2_O_2_ and O_2_^.−^ in the *Hph-1* mouse model	[79,80]
**CIPO (mitochondrial) and MNGIE**	Neuropathy (not all cases)	Genetic diseases associated with mitochondrial damage Variants in *LIG3* cause mitochondrial dysfunction and ↑mitochondrial O_2_^.−^	[81,82]
**Hirschsprung** **disease**	Neurochristopathy	Lack of enteric nervous system formation in model of intrauterine oxidative stress	[83]

Abbreviations: 4-HNE, 4-hydroxynonenal; 5-FU, 5-fluorouracil; 8-oxodG, 8-oxo-7,8-dihydro-2′-deoxyguanosine; CAT, catalase; CD, Crohn’s disease; CIPO, chronic intestinal pseudo-obstruction; COX, cyclooxygenases; CuZn-SOD, copper/zinc superoxide dismutase; CYP2E, cytochrome P450 2E1; GSH, glutathione; GSH-Px, glutathione peroxidase; GSSG-R, glutathione disulfide; HMGB1, high mobility group box-1; H_2_O_2_, hydrogen peroxide; HO-1, heme oxygenase-1; IBD, inflammatory bowel disease; ICC, interstitial cells of Cajal; IL-6, interleukin-6; iNOS, inducible nitric oxide synthase; LOX, lipoxygenase; LPS, lipopolysaccharide; MCP-1, monocyte chemoattractant protein-1; MDA, malondialdehyde; MNGIE, mitochondrial neurogastrointestinal encephalopathy; Mn-SOD, manganese superoxide dismutase; MPO, myeloperoxidase; NAC, N-acetyl cysteine; NAD, nicotinamide adenine dinucleotide; NEC, necrotizing enterocolitis; nNOS, neuronal nitric oxide synthase; NOS, nitric oxide synthase; NOX, NADPH oxidase; NOX5, NADPH oxidase-5; NSAIDs, nonsteroidal anti-inflammatory drugs; O_2_^.−^, superoxide; PGE2, prostaglandin E2; ROS, reactive oxygen species; SOD, superoxide dismutase; STZ, streptozocin; UC, ulcerative colitis; XOD, xanthine oxidase.

## References

[1] Fisher A.B. (2009). Redox signaling across cell membranes. Antioxid. Redox Signal..

[2] Valko M., Leibfritz D., Moncol J., Cronin M.T., Mazur M., Telser J. (2007). Free radicals and antioxidants in normal physiological functions and human disease. Int. J. Biochem. Cell Biol..

[3] Forman H.J., Zhang H. (2021). Targeting oxidative stress in disease: Promise and limitations of antioxidant therapy. Nat. Rev. Drug Discov..

[4] Li R., Jia Z., Trush M.A. (2016). Defining ROS in biology and medicine. React. Oxyg. Species.

[5] Al Ghouleh I., Khoo N.K., Knaus U.G., Griendling K.K., Touyz R.M., Thannickal V.J., Barchowsky A., Nauseef W.M., Kelley E.E., Bauer P.M. (2011). Oxidases and peroxidases in cardiovascular and lung disease: New concepts in reactive oxygen species signaling. Free Radic. Biol. Med..

[6] Rada B., Leto T.L. (2008). Oxidative innate immune defenses by Nox/Duox family NADPH oxidases. Contrib. Microbiol..

[7] Soldati T., Neyrolles O. (2012). Mycobacteria and the intraphagosomal environment: Take it with a pinch of salt(s)!. Traffic.

[8] Sanders K.M., Ward S.M. (2019). Nitric oxide and its role as a non-adrenergic, non-cholinergic inhibitory neurotransmitter in the gastrointestinal tract. Br. J. Pharmacol..

[9] Sies H. (2015). Oxidative stress: A concept in redox biology and medicine. Redox Biol..

[10] Sies H. (2021). Oxidative eustress: On constant alert for redox homeostasis. Redox Biol..

[11] Poprac P., Jomova K., Simunkova M., Kollar V., Rhodes C.J., Valko M. (2017). Targeting Free Radicals in Oxidative Stress-Related Human Diseases. Trends Pharmacol. Sci..

[12] Cho K.J., Seo J.M., Kim J.H. (2011). Bioactive lipoxygenase metabolites stimulation of NADPH oxidases and reactive oxygen species. Mol. Cells.

[13] Dan Dunn J., Alvarez L.A.J., Zhang X., Soldati T. (2015). Reactive oxygen species and mitochondria: A nexus of cellular homeostasis. Redox Biol..

[14] Chen Y., Azad M.B., Gibson S.B. (2009). Superoxide is the major reactive oxygen species regulating autophagy. Cell Death Differ..

[15] Ahmad R., Hussain A., Ahsan H. (2019). Peroxynitrite: Cellular pathology and implications in autoimmunity. J. Immunoass. Immunochem..

[16] De Deken X., Corvilain B., Dumont J.E., Miot F. (2014). Roles of DUOX-mediated hydrogen peroxide in metabolism, host defense, and signaling. Antioxid. Redox Signal..

[17] Winterbourn C.C. (1995). Toxicity of iron and hydrogen peroxide: The Fenton reaction. Toxicol. Lett..

[18] Masella R., Di Benedetto R., Varì R., Filesi C., Giovannini C. (2005). Novel mechanisms of natural antioxidant compounds in biological systems: Involvement of glutathione and glutathione-related enzymes. J. Nutr. Biochem..

[19] Choi K.M., Gibbons S.J., Nguyen T.V., Stoltz G.J., Lurken M.S., Ordog T., Szurszewski J.H., Farrugia G. (2008). Heme oxygenase-1 protects interstitial cells of Cajal from oxidative stress and reverses diabetic gastroparesis. Gastroenterology.

[20] Choi K.M., Kashyap P.C., Dutta N., Stoltz G.J., Ordog T., Shea Donohue T., Bauer A.J., Linden D.R., Szurszewski J.H., Gibbons S.J. (2010). CD206-positive M2 macrophages that express heme oxygenase-1 protect against diabetic gastroparesis in mice. Gastroenterology.

[21] Otterbein L.E., Choi A.M. (2000). Heme oxygenase: Colors of defense against cellular stress. Am. J. Physiol. Lung Cell. Mol. Physiol..

[22] Ryter S.W., Tyrrell R.M. (2000). The heme synthesis and degradation pathways: Role in oxidant sensitivity. Heme oxygenase has both pro- and antioxidant properties. Free Radic. Biol. Med..

[23] Jiménez P., Piazuelo E., Sánchez M.T., Ortego J., Soteras F., Lanas A. (2005). Free radicals and antioxidant systems in reflux esophagitis and Barrett’s esophagus. World J. Gastroenterol..

[24] Fu X., Beer D.G., Behar J., Wands J., Lambeth D., Cao W. (2006). cAMP-response element-binding protein mediates acid-induced NADPH oxidase NOX5-S expression in Barrett esophageal adenocarcinoma cells. J. Biol. Chem..

[25] Ersoz N., Ozler M., Topal T., Uysal B., Poyrazoglu Y., Simsek K., Gocgeldi E., Korkmaz A. (2016). The effect of ozone treatment on experimental colon anastomosis in rats. Eur. Surg..

[26] Farias Rolim M., Riger C.J., Eleutherio E.C., da Fonseca Colão C., Cotta Pereira G., Schanaider A. (2007). Colonic healing after portal ischemia and reperfusion: An experimental study with oxidative stress biomarkers. Redox Rep..

[27] Luo J., Wu H., Yang Y., Jiang Y., Yuan J., Tong Q. (2021). Oxidative Stress Level as a Predictor of Anastomotic Leakage after Rectal Surgery. Mediat. Inflamm..

[28] Pfeifle V.A., Gros S.J., Frongia G., Schäfer K.H., Holland-Cunz S. (2017). Regenerative Capacity of the Enteric Nervous System after Ileoileal Anastomoses in a Rat Model. Eur. J. Pediatr. Surg..

[29] Poyrazoglu Y., Yigit T., Harlak A., Mentes O., Gorgulu S., Uzar A.I., Kozak O. (2011). Effects of prevention of oxidative and nitro-oxidative stress on experimental rat colon anastomosis using acetylcysteine, Ebselen and 1400w. Acta Chir. Belg..

[30] McQuade R.M., Carbone S.E., Stojanovska V., Rahman A., Gwynne R.M., Robinson A.M., Goodman C.A., Bornstein J.C., Nurgali K. (2016). Role of oxidative stress in oxaliplatin-induced enteric neuropathy and colonic dysmotility in mice. Br. J. Pharmacol..

[31] Rtibi K., Selmi S., Grami D., Sebai H., Amri M., Marzouki L. (2017). Irinotecan chemotherapy-induced intestinal oxidative stress: Underlying causes of disturbed mucosal water and electrolyte transport. Pathophysiology.

[32] Shiota A., Hada T., Baba T., Sato M., Yamanaka-Okumura H., Yamamoto H., Taketani Y., Takeda E. (2010). Protective effects of glycoglycerolipids extracted from spinach on 5-fluorouracil induced intestinal mucosal injury. J. Med. Investig..

[33] Krecsmarik M., Izbéki F., Bagyánszki M., Linke N., Bódi N., Kaszaki J., Katarova Z., Szabó Á., Fekete E., Wittmann T. (2006). Chronic ethanol exposure impairs neuronal nitric oxide synthase in the rat intestine. Alcohol. Clin. Exp. Res..

[34] Keshavarzian A., Farhadi A., Forsyth C.B., Rangan J., Jakate S., Shaikh M., Banan A., Fields J.Z. (2009). Evidence that chronic alcohol exposure promotes intestinal oxidative stress, intestinal hyperpermeability and endotoxemia prior to development of alcoholic steatohepatitis in rats. J. Hepatol..

[35] Forsyth C.B., Voigt R.M., Shaikh M., Tang Y., Cederbaum A.I., Turek F.W., Keshavarzian A. (2013). Role for intestinal CYP2E1 in alcohol-induced circadian gene-mediated intestinal hyperpermeability. Am. J. Physiol.-Gastrointest. Liver Physiol..

[36] Samak G., Gangwar R., Meena A.S., Rao R.G., Shukla P.K., Manda B., Narayanan D., Jaggar J.H., Rao R. (2016). Calcium Channels and Oxidative Stress Mediate a Synergistic Disruption of Tight Junctions by Ethanol and Acetaldehyde in Caco-2 Cell Monolayers. Sci. Rep..

[37] Matosevic P., Klepac-Pulanic T., Kinda E., Augustin G., Brcic I., Jakic-Razumovic J. (2015). Immunohistochemical expression of 8-oxo-7,8-dihydro-2′-deoxyguanosine in cytoplasm of tumour and adjacent normal mucosa cells in patients with colorectal cancer. World J. Surg. Oncol..

[38] Skrzydlewska E., Sulkowski S., Koda M., Zalewski B., Kanczuga-Koda L., Sulkowska M. (2005). Lipid peroxidation and antioxidant status in colorectal cancer. World J. Gastroenterol..

[39] Godlewski J. (2010). Morphological changes in the enteric nervous system caused by carcinoma of the human large intestine. Folia Histochem. Cytobiol..

[40] Zauszkiewicz-Pawlak A., Godlewski J., Kwiatkowski P., Kmiec Z. (2017). Ultrastructural characteristics of myenteric plexus in patients with colorectal cancer. Folia Histochem. Cytobiol..

[41] Shebani K.O., Stucchi A.F., McClung J.P., Beer E.R., LaMorte W.W., Becker J.M. (2000). Role of Stasis and Oxidative Stress in Ileal Pouch Inflammation. J. Surg. Res..

[42] Zhao C.-M., Myrvold H.E., Chen D. (2015). Reduced neurons in the ileum of proctocolectomized rat models. Med. Mol. Morphol..

[43] Sasaki M., Joh T. (2007). Oxidative stress and ischemia-reperfusion injury in gastrointestinal tract and antioxidant, protective agents. J. Clin. Biochem. Nutr..

[44] Rivera L.R., Poole D.P., Thacker M., Furness J.B. (2011). The involvement of nitric oxide synthase neurons in enteric neuropathies. Neurogastroenterol. Motil..

[45] Rivera L.R., Thacker M., Pontell L., Cho H.-J., Furness J.B. (2011). Deleterious effects of intestinal ischemia/reperfusion injury in the mouse enteric nervous system are associated with protein nitrosylation. Cell Tissue Res..

[46] Klinowski E., Broide E., Varsano R., Eshchar J., Scapa E. (1996). Superoxide dismutase activity in duodenal ulcer patients. Eur. J. Gastroenterol. Hepatol..

[47] Stein H.J., Esplugues J., Whittle B.J., Bauerfeind P., Hinder R.A., Blum A.L. (1989). Direct cytotoxic effect of oxygen radicals on the gastric mucosa. Surgery.

[48] Esplugues J.V., Whittle B.J. (1989). Gastric damage following local intra-arterial administration of reactive oxygen metabolites in the rat. Br. J. Pharmacol..

[49] Vaananen P.M., Meddings J.B., Wallace J.L. (1991). Role of oxygen-derived free radicals in indomethacin-induced gastric injury. Am. J. Physiol.-Gastrointest. Liver Physiol..

[50] Czajkowska M., Całka J. (2020). Neurochemistry of Enteric Neurons Following Prolonged Indomethacin Administration in the Porcine Duodenum. Front. Pharmacol..

[51] Rząp D., Czajkowska M., Całka J. (2020). Neurochemical Plasticity of nNOS-, VIP- and CART-Immunoreactive Neurons Following Prolonged Acetylsalicylic Acid Supplementation in the Porcine Jejunum. Int. J. Mol. Sci..

[52] Schoffen J.P.F., Santi Rampazzo A.P., Cirilo C.P., Zapater M.C.U., Vicentini F.A., Comar J.F., Bracht A., Natali M.R.M. (2014). Food restriction enhances oxidative status in aging rats with neuroprotective effects on myenteric neuron populations in the proximal colon. Exp. Gerontol..

[53] Thrasivoulou C., Soubeyre V., Ridha H., Giuliani D., Giaroni C., Michael G.J., Saffrey M.J., Cowen T. (2006). Reactive oxygen species, dietary restriction and neurotrophic factors in age-related loss of myenteric neurons. Aging Cell.

[54] Musa A.E., Shabeeb D., Alhilfi H.S.Q. (2019). Protective effect of melatonin against radiotherapy-induced small intestinal oxidative stress: Biochemical evaluation. Medicina.

[55] Voss U., Malipatlolla D.K., Patel P., Devarakonda S., Sjöberg F., Grandér R., Rascón A., Nyman M., Steineck G., Bull C. (2022). Irradiation Induces Tuft Cell Hyperplasia and Myenteric Neuronal Loss in the Absence of Dietary Fiber in a Mouse Model of Pelvic Radiotherapy. Gastroenterol. Insights.

[56] Storr H.L., Kind B., Parfitt D.A., Chapple J.P., Lorenz M., Koehler K., Huebner A., Clark A.J. (2009). Deficiency of ferritin heavy-chain nuclear import in triple a syndrome implies nuclear oxidative damage as the primary disease mechanism. Mol. Endocrinol..

[57] Prasad R., Kowalczyk J., Meimaridou E., Storr H., Metherell L. (2014). Oxidative stress and adrenocortical insufficiency. J. Endocrinol..

[58] Aceti A., Beghetti I., Martini S., Faldella G., Corvaglia L. (2018). Oxidative Stress and Necrotizing Enterocolitis: Pathogenetic Mechanisms, Opportunities for Intervention, and Role of Human Milk. Oxidative Med. Cell. Longev..

[59] Aydemir C., Dilli D., Uras N., Ulu H.O., Oguz S.S., Erdeve O., Dilmen U. (2011). Total oxidant status and oxidative stress are increased in infants with necrotizing enterocolitis. J. Pediatr. Surg..

[60] Perrone S., Tataranno M.L., Negro S., Cornacchione S., Longini M., Proietti F., Soubasi V., Benders M.J., Van Bel F., Buonocore G. (2012). May oxidative stress biomarkers in cord blood predict the occurrence of necrotizing enterocolitis in preterm infants?. J. Matern. Fetal Neonatal Med..

[61] Whitehouse J.S., Xu H., Shi Y., Noll L., Kaul S., Jones D.W., Pritchard K.A., Oldham K.T., Gourlay D.M. (2010). Mesenteric nitric oxide and superoxide production in experimental necrotizing enterocolitis. J. Surg. Res..

[62] Zhou Y., Yang J., Watkins D.J., Boomer L.A., Matthews M.A., Su Y., Besner G.E. (2013). Enteric nervous system abnormalities are present in human necrotizing enterocolitis: Potential neurotransplantation therapy. Stem Cell Res. Ther..

[63] Di S.-J., Wu S.-Y., Liu T.-J., Shi Y.-Y. (2022). Stem cell therapy as a promising strategy in necrotizing enterocolitis. Mol. Med..

[64] Silva K.D.A., Nunes J.P.S., Andrieux P., Brochet P., Almeida R.R., Kuramoto Takara A.C.K., Pereira N.B., Abel L., Cobat A., Zaniratto R.C.F. (2022). Chagas Disease Megaesophagus Patients Carrying Variant *MRPS18B* P260A Display Nitro-Oxidative Stress and Mitochondrial Dysfunction in Response to IFN-γ; Stimulus. Biomedicines.

[65] Ricci M.F., Béla S.R., Moraes M.M., Bahia M.T., Mazzeti A.L., Oliveira A.C.S., Andrade L.O., Radí R., Piacenza L., Arantes R.M.E. (2020). Neuronal Parasitism, Early Myenteric Neurons Depopulation and Continuous Axonal Networking Damage as Underlying Mechanisms of the Experimental Intestinal Chagas’ Disease. Front. Cell. Infect. Microbiol..

[66] Bhor V.M., Raghuram N., Sivakami S. (2004). Oxidative damage and altered antioxidant enzyme activities in the small intestine of streptozotocin-induced diabetic rats. Int. J. Biochem. Cell Biol..

[67] Rivoira M., Rodríguez V., López M.P., Tolosa de Talamoni N. (2015). Time dependent changes in the intestinal Ca^2+^ absorption in rats with type I diabetes mellitus are associated with alterations in the intestinal redox state. Biochim. Biophys. Acta (BBA) Mol. Basis Dis..

[68] Chandrasekharan B., Anitha M., Blatt R., Shahnavaz N., Kooby D., Staley C., Mwangi S., Jones D.P., Sitaraman S.V., Srinivasan S. (2011). Colonic motor dysfunction in human diabetes is associated with enteric neuronal loss and increased oxidative stress. Neurogastroenterol. Motil..

[69] Brown I.A.M., McClain J.L., Watson R.E., Patel B.A., Gulbransen B.D. (2016). Enteric Glia Mediate Neuron Death in Colitis Through Purinergic Pathways That Require Connexin-43 and Nitric Oxide. Cell. Mol. Gastroenterol. Hepatol..

[70] D’Inca R., Cardin R., Benazzato L., Angriman I., Martines D., Sturniolo G.C. (2004). Oxidative DNA damage in the mucosa of ulcerative colitis increases with disease duration and dysplasia. Inflamm. Bowel Dis..

[71] Koutroubakis I.E., Malliaraki N., Dimoulios P.D., Karmiris K., Castanas E., Kouroumalis E.A. (2004). Decreased total and corrected antioxidant capacity in patients with inflammatory bowel disease. Dig. Dis. Sci..

[72] Lam G., Apostolopoulos V., Zulli A., Nurgali K. (2015). NADPH oxidases and inflammatory bowel disease. Curr. Med. Chem..

[73] Lih-Brody L., Powell S.R., Collier K.P., Reddy G.M., Cerchia R., Kahn E., Weissman G.S., Katz S., Floyd R.A., McKinley M.J. (1996). Increased oxidative stress and decreased antioxidant defenses in mucosa of inflammatory bowel disease. Dig. Dis. Sci..

[74] Pereira C., Coelho R., Grácio D., Dias C., Silva M., Peixoto A., Lopes P., Costa C., Teixeira J.P., Macedo G. (2016). DNA Damage and Oxidative DNA Damage in Inflammatory Bowel Disease. J. Crohn’s Colitis.

[75] Piechota-Polanczyk A., Fichna J. (2014). Review article: The role of oxidative stress in pathogenesis and treatment of inflammatory bowel diseases. Naunyn-Schmiedeberg’s Arch. Pharmacol..

[76] Stavely R., Sahakian L., Filippone R.T., Stojanovska V., Bornstein J.C., Sakkal S., Nurgali K. (2022). Oxidative Stress-Induced HMGB1 Translocation in Myenteric Neurons Contributes to Neuropathy in Colitis. Biomolecules.

[77] Farro G., Gomez-Pinilla P.J., Di Giovangiulio M., Stakenborg N., Auteri M., Thijs T., Depoortere I., Matteoli G., Boeckxstaens G.E. (2016). Smooth muscle and neural dysfunction contribute to different phases of murine postoperative ileus. Neurogastroenterol. Motil..

[78] De Backer O., Elinck E., Blanckaert B., Leybaert L., Motterlini R., Lefebvre R.A. (2009). Water-soluble CO-releasing molecules reduce the development of postoperative ileus via modulation of MAPK/HO-1 signalling and reduction of oxidative stress. Gut.

[79] Dieler R., Schröder J.M. (1989). Myenteric plexus neuropathy in infantile hypertrophic pyloric stenosis. Acta Neuropathol..

[80] Welsh C., Shifrin Y., Pan J., Belik J. (2014). Infantile hypertrophic pyloric stenosis (IHPS): A study of its pathophysiology utilizing the newborn hph-1 mouse model of the disease. Am. J. Physiol.-Gastrointest. Liver Physiol..

[81] Bonora E., Chakrabarty S., Kellaris G., Tsutsumi M., Bianco F., Bergamini C., Ullah F., Isidori F., Liparulo I., Diquigiovanni C. (2021). Biallelic variants in *LIG3* cause a novel mitochondrial neurogastrointestinal encephalomyopathy. Brain.

[82] Bianco F., Lattanzio G., Lorenzini L., Mazzoni M., Clavenzani P., Calzà L., Giardino L., Sternini C., Costanzini A., Bonora E. (2022). Enteric Neuromyopathies: Highlights on Genetic Mechanisms Underlying Chronic Intestinal Pseudo-Obstruction. Biomolecules.

[83] Zhou L., Wang B., Xie H., Du C., Tang J., Tang W. (2022). Intrauterine exposure to oxidative stress induces caspase-1-dependent enteric nerve cell pyroptosis. Pediatr. Surg. Int..

[84] Conklin K.A. (2004). Chemotherapy-associated oxidative stress: Impact on chemotherapeutic effectiveness. Integr. Cancer Ther..

[85] Bhattacharyya A., Chattopadhyay R., Mitra S., Crowe S.E. (2014). Oxidative stress: An essential factor in the pathogenesis of gastrointestinal mucosal diseases. Physiol. Rev..

[86] Orlikova B., Legrand N., Panning J., Dicato M., Diederich M. (2014). Anti-inflammatory and anticancer drugs from nature. Cancer Treat. Res..

[87] Zeng D., Wang Y., Chen Y., Li D., Li G., Xiao H., Hou J., Wang Z., Hu L., Wang L. (2021). Angelica Polysaccharide Antagonizes 5-FU-Induced Oxidative Stress Injury to Reduce Apoptosis in the Liver Through Nrf2 Pathway. Front. Oncol..

[88] Yang H., Villani R.M., Wang H., Simpson M.J., Roberts M.S., Tang M., Liang X. (2018). The role of cellular reactive oxygen species in cancer chemotherapy. J. Exp. Clin. Cancer Res..

[89] Conklin K.A. (2000). Dietary antioxidants during cancer chemotherapy: Impact on chemotherapeutic effectiveness and development of side effects. Nutr. Cancer.

[90] Bai G.W., Han D.Y., Yang Q.Y., Xie Y., Guo Z.X., Zhou W.L., Deng C.H., Sun X.Z. (2019). Oxidative stress induces damage to epididymal epithelial tight junction protein ZO-1 and impairs epididymal function in varicocele rats. Natl. J. Androl..

[91] Cao S.S. (2018). Cellular Stress Responses and Gut Microbiota in Inflammatory Bowel Disease. Gastroenterol. Res. Pr..

[92] Vivarelli S., Salemi R., Candido S., Falzone L., Santagati M., Stefani S., Torino F., Banna G.L., Tonini G., Libra M. (2019). Gut Microbiota and Cancer: From Pathogenesis to Therapy. Cancers.

[93] McQuade R.M., Stojanovska V., Abalo R., Bornstein J.C., Nurgali K. (2016). Chemotherapy-Induced Constipation and Diarrhea: Pathophysiology, Current and Emerging Treatments. Front. Pharmacol..

[94] Escalante J., McQuade R.M., Stojanovska V., Nurgali K. (2017). Impact of chemotherapy on gastrointestinal functions and the enteric nervous system. Maturitas.

[95] Nurgali K., Jagoe R.T., Abalo R. (2018). Adverse effects of cancer chemotherapy: Anything new to improve tolerance and reduce sequelae?. Front. Pharmacol..

[96] Van Vliet M.J., Tissing W.J.E., Dun C.A.J., Meessen N.E.L., Kamps W.A., de Bont E.S.J.M., Harmsen H.J.M. (2009). Chemotherapy Treatment in Pediatric Patients with Acute Myeloid Leukemia Receiving Antimicrobial Prophylaxis Leads to a Relative Increase of Colonization with Potentially Pathogenic Bacteria in the Gut. Clin. Infect. Dis..

[97] Pulito C., Cristaudo A., Porta C.L., Zapperi S., Blandino G., Morrone A., Strano S. (2020). Oral mucositis: The hidden side of cancer therapy. J. Exp. Clin. Cancer Res..

[98] Sonis S.T. (2004). The pathobiology of mucositis. Nat. Rev. Cancer.

[99] Al-Asmari A.K., Khan A.Q., Al-Asmari S.A., Al-Rawi A., Al-Omani S. (2016). Alleviation of 5-fluorouracil-induced intestinal mucositis in rats by vitamin E via targeting oxidative stress and inflammatory markers. J. Complement. Integr. Med..

[100] Was H., Borkowska A., Bagues A., Tu L., Liu J.Y., Lu Z., Rudd J.A., Nurgali K., Abalo R. (2022). Mechanisms of chemotherapy-induced neurotoxicity. Front. Pharmacol..

[101] Liu R., Bian Y., Liu L., Liu L., Liu X., Ma S. (2022). Molecular pathways associated with oxidative stress and their potential applications in radiotherapy. Int. J. Mol. Med..

[102] Richardson R.B., Harper M.E. (2016). Mitochondrial stress controls the radiosensitivity of the oxygen effect: Implications for radiotherapy. Oncotarget.

[103] Galati G., Tafazoli S., Sabzevari O., Chan T.S., O’Brien P.J. (2002). Idiosyncratic NSAID drug induced oxidative stress. Chem.-Biol. Interact..

[104] Carrasco-Pozo C., Gotteland M., Speisky H. (2011). Apple peel polyphenol extract protects against indomethacin-induced damage in Caco-2 cells by preventing mitochondrial complex I inhibition. J. Agric. Food Chem..

[105] Nagano Y., Matsui H., Shimokawa O., Hirayama A., Tamura M., Nakamura Y., Kaneko T., Rai K., Indo H., Majima H. (2012). Rebamipide attenuates nonsteroidal anti-inflammatory drugs (NSAID) induced lipid peroxidation by the manganese superoxide dismutase (MnSOD) overexpression in gastrointestinal epithelial cells. J. Physiol. Pharmacol..

[106] Uc A., Vasiliauskas E., Piccoli D.A., Flores A.F., Di Lorenzo C., Hyman P.E. (1997). Chronic intestinal pseudoobstruction associated with fetal alcohol syndrome. Dig. Dis. Sci..

[107] De Vries D.K., Kortekaas K.A., Tsikas D., Wijermars L.G., van Noorden C.J., Suchy M.T., Cobbaert C.M., Klautz R.J., Schaapherder A.F., Lindeman J.H. (2013). Oxidative damage in clinical ischemia/reperfusion injury: A reappraisal. Antioxid. Redox Signal..

[108] Amaya Y., Yamazaki K., Sato M., Noda K., Nishino T., Nishino T. (1990). Proteolytic conversion of xanthine dehydrogenase from the NAD-dependent type to the O2-dependent type. Amino acid sequence of rat liver xanthine dehydrogenase and identification of the cleavage sites of the enzyme protein during irreversible conversion by trypsin. J. Biol. Chem..

[109] Chung H.Y., Baek B.S., Song S.H., Kim M.S., Huh J.I., Shim K.H., Kim K.W., Lee K.H. (1997). Xanthine dehydrogenase/xanthine oxidase and oxidative stress. Age.

[110] Bauer A.J., Boeckxstaens G.E. (2004). Mechanisms of postoperative ileus. Neurogastroenterol. Motil..

[111] Kalff J.C., Schraut W.H., Simmons R.L., Bauer A.J. (1998). Surgical manipulation of the gut elicits an intestinal muscularis inflammatory response resulting in postsurgical ileus. Ann. Surg..

[112] Matsumoto K., Kawanaka H., Hori M., Kusamori K., Utsumi D., Tsukahara T., Amagase K., Horie S., Yamamoto A., Ozaki H. (2018). Role of transient receptor potential melastatin 2 in surgical inflammation and dysmotility in a mouse model of postoperative ileus. Am. J. Physiol.-Gastrointest. Liver Physiol..

[113] Smeitink J.A., Zeviani M., Turnbull D.M., Jacobs H.T. (2006). Mitochondrial medicine: A metabolic perspective on the pathology of oxidative phosphorylation disorders. Cell Metab..

[114] Schill E.M., Lake J.I., Tusheva O.A., Nagy N., Bery S.K., Foster L., Avetisyan M., Johnson S.L., Stenson W.F., Goldstein A.M. (2016). Ibuprofen slows migration and inhibits bowel colonization by enteric nervous system precursors in zebrafish, chick and mouse. Dev. Biol..

[115] Torres J., Mehandru S., Colombel J.-F., Peyrin-Biroulet L. (2017). Crohn’s Disease. Lancet.

[116] Ungaro R., Mehandru S., Allen P.B., Peyrin-Biroulet L., Colombel J.-F. (2017). Ulcerative Colitis. Lancet.

[117] Hancock L., Windsor A.C., Mortensen N.J. (2006). Inflammatory bowel disease: The view of the surgeon. Color. Dis..

[118] Lakhan S.E., Kirchgessner A. (2010). Neuroinflammation in inflammatory bowel disease. J. Neuroinflammation.

[119] Sahakian L., Filippone R.T., Stavely R., Robinson A.M., Yan X.S., Abalo R., Eri R., Bornstein J.C., Kelley M.R., Nurgali K. (2021). Inhibition of APE1/Ref-1 redox signaling alleviates intestinal dysfunction and damage to myenteric neurons in a mouse model of spontaneous chronic colitis. Inflamm. Bowel Dis..

[120] Tian T., Wang Z., Zhang J. (2017). Pathomechanisms of oxidative stress in inflammatory bowel disease and potential antioxidant therapies. Oxidative Med. Cell. Longev..

[121] Rezaie A., Parker R.D., Abdollahi M. (2007). Oxidative stress and pathogenesis of inflammatory bowel disease: An epiphenomenon or the cause?. Dig. Dis. Sci..

[122] Hackam D.J., Sodhi C.P., Good M. (2019). New insights into necrotizing enterocolitis: From laboratory observation to personalized prevention and treatment. J. Pediatr. Surg..

[123] Nogueira N.P., Saraiva F.M.S., Sultano P.E., Cunha P.R.B.B., Laranja G.A.T., Justo G.A., Sabino K.C.C., Coelho M.G.P., Rossini A., Atella G.C. (2015). Proliferation and Differentiation of *Trypanosoma cruzi* inside Its Vector Have a New Trigger: Redox Status. PLoS ONE.

[124] Weimers P., Munkholm P. (2018). The Natural History of IBD: Lessons Learned. Curr. Treat. Options Gastroenterol..

[125] Yalchin M., Baker A.M., Graham T.A., Hart A. (2021). Predicting Colorectal Cancer Occurrence in IBD. Cancers.

[126] Carini F., Mazzola M., Rappa F., Jurjus A., Geagea A.G., Al Kattar S., Bou-Assi T., Jurjus R., Damiani P., Leone A. (2017). Colorectal carcinogenesis: Role of oxidative stress and antioxidants. Anticancer Res..

[127] Peng D., Zaika A., Que J., El-Rifai W. (2021). The antioxidant response in Barrett’s tumorigenesis: A double-edged sword. Redox Biol..

[128] Peng D., Hu T., Soutto M., Belkhiri A., Zaika A., El-Rifai W. (2014). Glutathione peroxidase 7 has potential tumour suppressor functions that are silenced by location-specific methylation in oesophageal adenocarcinoma. Gut.

[129] Peng D.F., Razvi M., Chen H., Washington K., Roessner A., Schneider-Stock R., El-Rifai W. (2009). DNA hypermethylation regulates the expression of members of the Mu-class glutathione S-transferases and glutathione peroxidases in Barrett’s adenocarcinoma. Gut.

[130] Martínez Leo E.E., Peñafiel A.M., Hernández Escalante V.M., Cabrera Araujo Z.M. (2021). Ultra-processed diet, systemic oxidative stress, and breach of immunologic tolerance. Nutrition.

[131] Stavely R., Abalo R., Nurgali K. (2020). Targeting Enteric Neurons and Plexitis for the Management of Inflammatory Bowel Disease. Curr. Drug Targets.

[132] Furness J.B. (2006). The Enteric Nervous System.

[133] Schneider S., Wright C.M., Heuckeroth R.O. (2019). Unexpected Roles for the Second Brain: Enteric Nervous System as Master Regulator of Bowel Function. Annu. Rev. Physiol..

[134] Furness J.B. (2012). The Enteric Nervous System and Neurogastroenterology. Nat. Rev. Gastroenterol. Hepatol..

[135] Goldstein A.M., Thapar N., Karunaratne T.B., De Giorgio R. (2016). Clinical aspects of neurointestinal disease: Pathophysiology, diagnosis, and treatment. Dev. Biol..

[136] Pellegrini C., D’Antongiovanni V., Miraglia F., Rota L., Benvenuti L., Di Salvo C., Testa G., Capsoni S., Carta G., Antonioli L. (2022). Enteric α-synuclein impairs intestinal epithelial barrier through caspase-1-inflammasome signaling in Parkinson’s disease before brain pathology. NPJ Park. Dis..

[137] Rota L., Pellegrini C., Benvenuti L., Antonioli L., Fornai M., Blandizzi C., Cattaneo A., Colla E. (2019). Constipation, deficit in colon contractions and alpha-synuclein inclusions within the colon precede motor abnormalities and neurodegeneration in the central nervous system in a mouse model of alpha-synucleinopathy. Transl. Neurodegener..

[138] Friedman J. (2011). Why is the nervous system vulnerable to oxidative stress. Oxidative Stress and Free Radical Damage in Neurology.

[139] Wada-Takahashi S., Tamura K. (2000). Actions of reactive oxygen species on AH/type 2 myenteric neurons in guinea pig distal colon. Am. J. Physiol.-Gastrointest. Liver Physiol..

[140] Roberts J.A., Durnin L., Sharkey K.A., Mutafova-Yambolieva V.N., Mawe G.M. (2013). Oxidative stress disrupts purinergic neuromuscular transmission in the inflamed colon. J. Physiol..

[141] McQuade R.M., Stojanovska V., Stavely R., Timpani C., Petersen A.C., Abalo R., Bornstein J.C., Rybalka E., Nurgali K. (2018). Oxaliplatin-induced enteric neuronal loss and intestinal dysfunction is prevented by co-treatment with BGP-15. Br. J. Pharmacol..

[142] Abdo H., Derkinderen P., Gomes P., Chevalier J., Aubert P., Masson D., Galmiche J.P., Vanden Berghe P., Neunlist M., Lardeux B. (2010). Enteric glial cells protect neurons from oxidative stress in part via reduced glutathione. Faseb J..

[143] Brown I.A.M., Gulbransen B.D. (2018). The antioxidant glutathione protects against enteric neuron death in situ, but its depletion is protective during colitis. Am. J. Physiol. Gastrointest. Liver Physiol..

[144] Linden D., Couvrette J., Ciolino A., McQuoid C., Blaszyk H., Sharkey K., Mawe G. (2005). Indiscriminate loss of myenteric neurones in the TNBS-inflamed guinea-pig distal colon. Neurogastroenterol. Motil..

[145] Winston J.H., Li Q., Sarna S.K. (2013). Paradoxical regulation of ChAT and nNOS expression in animal models of Crohn’s colitis and ulcerative colitis. Am. J. Physiol. Gastrointest. Liver Physiol..

[146] Stavely R., Robinson A.M., Miller S., Boyd R., Sakkal S., Nurgali K. (2015). Human adult stem cells derived from adipose tissue and bone marrow attenuate enteric neuropathy in the guinea-pig model of acute colitis. Stem Cell Res. Ther..

[147] Stavely R., Robinson A.M., Miller S., Boyd R., Sakkal S., Nurgali K. (2015). Allogeneic guinea pig mesenchymal stem cells ameliorate neurological changes in experimental colitis. Stem Cell Res. Ther..

[148] Bubenheimer R.K., Brown I.A.M., Fried D.E., McClain J.L., Gulbransen B.D. (2016). Sirtuin-3 Is Expressed by Enteric Neurons but It Does not Play a Major Role in Their Regulation of Oxidative Stress. Front. Cell. Neurosci..

[149] Nurgali K., Qu Z., Hunne B., Thacker M., Pontell L., Furness J.B. (2011). Morphological and functional changes in guinea-pig neurons projecting to the ileal mucosa at early stages after inflammatory damage. J. Physiol..

[150] Qu Z.-D., Thacker M., Castelucci P., Bagyánszki M., Epstein M.L., Furness J.B. (2008). Immunohistochemical analysis of neuron types in the mouse small intestine. Cell Tissue Res..

[151] Wafai L., Taher M., Jovanovska V., Bornstein J.C., Dass C.R., Nurgali K. (2013). Effects of oxaliplatin on mouse myenteric neurons and colonic motility. Front. Neurosci..

[152] Bagyánszki M., Krecsmarik M., De Winter B.Y., De Man J.G., Fekete E., Pelckmans P.A., Adriaensen D., Kroese A.B., Van Nassauw L., Timmermans J.P. (2010). Chronic alcohol consumption affects gastrointestinal motility and reduces the proportion of neuronal NOS-immunoreactive myenteric neurons in the murine jejunum. Anat. Record.

[153] Izbéki F., Wittman T., Rosztóczy A., Linke N., Bódi N., Fekete E., Bagyánszki M. (2008). Immediate insulin treatment prevents gut motility alterations and loss of nitrergic neurons in the ileum and colon of rats with streptozotocin-induced diabetes. Diabetes Res. Clin. Pract..

[154] Bódi N., Szalai Z., Bagyánszki M. (2019). Nitrergic Enteric Neurons in Health and Disease-Focus on Animal Models. Int. J. Mol. Sci..

[155] Voukali E., Shotton H., Lincoln J. (2011). Selective responses of myenteric neurons to oxidative stress and diabetic stimuli. Neurogastroenterol. Motil..

[156] Berghe P.V., Kenyon J.L., Smith T.K. (2002). Mitochondrial Ca^2+^ uptake regulates the excitability of myenteric neurons. J. Neurosci..

[157] Aon M.A., Cortassa S., O’Rourke B. (2010). Redox-optimized ROS balance: A unifying hypothesis. Biochim. Biophys. Acta.

[158] Jia J., Jin H., Nan D., Yu W., Huang Y. (2021). New insights into targeting mitochondria in ischemic injury. Apoptosis.

[159] Kwak D.J., Kwak S.D., Gauda E.B. (2006). The effect of hyperoxia on reactive oxygen species (ROS) in rat petrosal ganglion neurons during development using organotypic slices. Pediatr. Res..

[160] D’Agostino D.P., Putnam R.W., Dean J.B. (2007). Superoxide (O_2_^.−^) production in CA1 neurons of rat hippocampal slices exposed to graded levels of oxygen. J. Neurophysiol..

[161] Murphy M.P. (2009). How mitochondria produce reactive oxygen species. Biochem. J..

[162] Matott M.P., Ciarlone G.E., Putnam R.W., Dean J.B. (2014). Normobaric hyperoxia (95% O_2_) stimulates CO_2_-sensitive and CO_2_-insensitive neurons in the caudal solitary complex of rat medullary tissue slices maintained in 40% O_2_. Neuroscience.

[163] Chang E., Hornick K., Fritz K.I., Mishra O.P., Delivoria-Papadopoulos M. (2007). Effect of hyperoxia on cortical neuronal nuclear function and programmed cell death mechanisms. Neurochem. Res..

[164] Wang J., Xiao J., Meng X., Chu X., Zhuansun D.D., Xiong B., Feng J. (2021). NOX5 is expressed aberrantly but not a critical pathogenetic gene in Hirschsprung disease. BMC Pediatr..

[165] Görlach A., Bertram K., Hudecova S., Krizanova O. (2015). Calcium and ROS: A mutual interplay. Redox Biol..

[166] Kourie J.I. (1998). Interaction of reactive oxygen species with ion transport mechanisms. Am. J. Physiol.-Cell Physiol..

[167] Patergnani S., Morciano G., Carinci M., Leo S., Pinton P., Rimessi A. (2022). The “mitochondrial stress responses”: The “Dr. Jekyll and Mr. Hyde” of neuronal disorders. Neural Regen. Res..

[168] Hirano M., Nishigaki Y., Martí R. (2004). Mitochondrial neurogastrointestinal encephalomyopathy (MNGIE): A disease of two genomes. Neurologist.

[169] Sundaram U., Hassanain H., Suntres Z., Yu J.G., Cooke H.J., Guzman J., Christofi F.L. (2003). Rabbit chronic ileitis leads to up-regulation of *adenosine A1/A3* gene products, oxidative stress, and immune modulation. Biochem. Pharmacol..

[170] Shi X.-Z., Winston J.H., Sarna S.K. (2010). Differential immune and genetic responses in rat models of Crohn’s colitis and ulcerative colitis. Am. J. Physiol.-Gastrointest. Liver Physiol..

[171] Bianco F., Bonora E., Natarajan D., Vargiolu M., Thapar N., Torresan F., Giancola F., Boschetti E., Volta U., Bazzoli F. (2016). Prucalopride exerts neuroprotection in human enteric neurons. Am. J. Physiol.-Gastrointest. Liver Physiol..

[172] Korsak K., Silva A.T., Saffrey M.J. (2012). Differing effects of NT-3 and GDNF on dissociated enteric ganglion cells exposed to hydrogen peroxide in vitro. Neurosci. Lett..

[173] Lourenssen S., Miller K.G., Blennerhassett M.G. (2009). Discrete responses of myenteric neurons to structural and functional damage by neurotoxins in vitro. Am. J. Physiol.-Gastrointest. Liver Physiol..

[174] Pouokam E., Rehn M., Diener M. (2009). Effects of H_2_O_2_ at rat myenteric neurones in culture. Eur. J. Pharmacol..

[175] Gong G., Xiang L., Yuan L., Hu L., Wu W., Cai L., Yin L., Dong H. (2014). Protective effect of glycyrrhizin, a direct HMGB1 inhibitor, on focal cerebral ischemia/reperfusion-induced inflammation, oxidative stress, and apoptosis in rats. PLoS ONE.

[176] Balasubramaniam A., Li G., Ramanathan A., Mwangi S.M., Hart C.M., Arbiser J.L., Srinivasan S. (2022). SIRT3 activation promotes enteric neurons survival and differentiation. Sci. Rep..

[177] Sahakian L., McQuade R., Stavely R., Robinson A., Filippone R.T., Hassanzadeganroudsari M., Eri R., Abalo R., Bornstein J.C., Kelley M.R. (2022). Molecular Targets to Alleviate Enteric Neuropathy and Gastrointestinal Dysfunction. Adv. Exp. Med. Biol..

[178] Belkind-Gerson J., Graham H.K., Reynolds J., Hotta R., Nagy N., Cheng L., Kamionek M., Shi H.N., Aherne C.M., Goldstein A.M. (2017). Colitis promotes neuronal differentiation of Sox2+ and PLP1+ enteric cells. Sci. Rep..

[179] Belkind-Gerson J., Hotta R., Nagy N., Thomas A.R., Graham H., Cheng L., Solorzano J., Nguyen D., Kamionek M., Dietrich J. (2015). Colitis Induces Enteric Neurogenesis Through a 5-HT4–dependent Mechanism. Inflamm. Bowel Dis..

[180] Huang T.-T., Zou Y., Corniola R. (2012). Oxidative stress and adult neurogenesis—Effects of radiation and superoxide dismutase deficiency. Seminars in Cell & Developmental Biology.

